# Characterizing the microbiota of cleft lip and palate patients: a comprehensive review

**DOI:** 10.3389/fcimb.2023.1159455

**Published:** 2023-04-18

**Authors:** Elizabeth Gershater, Yuan Liu, Binglan Xue, Min Kyung Shin, Hyun Koo, Zhong Zheng, Chenshuang Li

**Affiliations:** ^1^ Department of Biology, Muhlenberg College, Allentown, PA, United States; ^2^ Biofilm Research Laboratories, Levy Center for Oral Health, School of Dental Medicine, University of Pennsylvania, Philadelphia, PA, United States; ^3^ Department of Preventive and Restorative Sciences, School of Dental Medicine, University of Pennsylvania, Philadelphia, PA, United States; ^4^ School of Dental Medicine, University of Pennsylvania, Philadelphia, PA, United States; ^5^ Department of Orthodontics, School of Dental Medicine, University of Pennsylvania, Philadelphia, PA, United States; ^6^ Center for Innovation & Precision Dentistry, School of Dental Medicine and School of Engineering & Applied Sciences, University of Pennsylvania, Philadelphia, PA, United States; ^7^ David Geffen School of Medicine, University of California, Los Angeles, Los Angeles, CA, United States

**Keywords:** cleft lip, cleft palate, biofilm, oral microbiome, bacteria, fungus, virus

## Abstract

Orofacial cleft disorders, including cleft lip and/or palate (CL/P), are one of the most frequently-occurring congenital disorders worldwide. The health issues of patients with CL/P encompass far more than just their anatomic anomaly, as patients with CL/P are prone to having a high incidence of infectious diseases. While it has been previously established that the oral microbiome of patients with CL/P differs from that of unaffected patients, the exact nature of this variance, including the relevant bacterial species, has not been fully elucidated; likewise, examination of anatomic locations besides the cleft site has been neglected. Here, we intended to provide a comprehensive review to highlight the significant microbiota differences between CL/P patients and healthy subjects in various anatomic locations, including the teeth inside and adjacent to the cleft, oral cavity, nasal cavity, pharynx, and ear, as well as bodily fluids, secretions, and excretions. A number of bacterial and fungal species that have been proven to be pathogenic were found to be prevalently and/or specifically detected in CL/P patients, which can benefit the development of CL/P-specific microbiota management strategies.

## 1 Introduction

Fusion of particular orofacial structures during early gestation is required for proper development of the upper lip and jaw (Hammond and Dixon, 2022). Failure of this process results in an orofacial cleft, which manifests as a gap in the tissue of the upper lip, the palate, or both (Mossey et al., 2009). Affecting around 1 out of 700 live births, cleft lip and/or palate (CL/P) is one of the most common congenital craniofacial disorders (Candotto et al., 2019).

CL/P significantly reduces patients’ quality of life (Mossey et al., 2009). Firstly, some patients have an abnormal nasal bone structure and shape (Tibesar et al., 2009), which may be accompanied by malformations of oral muscles (Marazita, 2007; Li et al., 2019). Thus, CL/P infants may not feed properly after birth due to impaired aspiration and deglutition (Duarte et al., 2016), hindering their overall growth and development (Bessell et al., 2011). In addition, oral muscle maldevelopment may lead to infection of the Eustachian tubes and subsequent deafness, contributing to communication difficulties (Vyas et al., 2020). Consequently, verbal communication is specifically challenging for CL/P patients, and their interpersonal relations can be damaged by their speech and outward presentation (Mossey et al., 2009). Moreover, people born with CL/P may continue to have a poor impression of their face and desire to alter certain aspects of their appearance even as adults (Meyer-Marcotty and Stellzig-Eisenhauer, 2009).

Unsurprisingly, various dental conditions, such as enamel hypoplasia, asymmetrical development of the dentition, and microdontia, are also associated with CL/P (Van Dyck et al., 2019; Vyas et al., 2020). Noticeably, CL/P-associated dental health issues encompass far more than the anatomic anomaly itself. For example, CL/P patients may experience delayed tooth development or eruption, particularly of the primary molars, generally by half a year compared to their non-CL/P peers (Van Dyck et al., 2019; Vyas et al., 2020). Moreover, compared to their healthy peers, children with CL/P present much more severe signs of poor oral health, develop dental caries at a higher rate, and have a higher risk of Eustachian tube infections (Bessell et al., 2011; Zhang et al., 2016; Rodrigues et al., 2019), all of which are associated with bacterial infections. Furthermore, CL/P patients harbor different oral microbiota compared to individuals with normal orofacial development (Zhang et al., 2016; Funahashi et al., 2019), which may potentially be attributed to the presence of an oronasal fistula that allows bacterial transmission from the nose to the mouth (Tuna et al., 2008). Another potential cause of the aberrant oral microbiomes seen in CL/P patients is the existence of a cleft (even after surgical correction, a residual cleft may exist) that markedly increases the difficulty of toothbrushing, possibly due to inaccessibility of the cleft region or unwillingness to brush because of a misconception that toothbrushing risks damaging the repaired area (Tuna et al., 2008; Rodrigues et al., 2019). In any case, the aberrant oral microbiomes that result from the development and surgical correction of CL/P can, in turn, have significant implications for the oral health of CL/P patients as well as the bacterial composition and condition of other anatomic sites (Tuna et al., 2008; Zhang et al., 2016; Funahashi et al., 2019). This review aims to further elucidate the different microbiota in various niches that differ between CL/P patients and healthy persons based on currently available publications, to benefit the development of CL/P-specific microbiota management strategies.

## 2 Original article searching and selection

To include all the available original studies on CL/P-related microbiomes, this study was conducted following the 2020 Preferred Reporting Items for Systematic Reviews and Meta-Analyses (PRISMA) guidelines. The article search was carried out in August 2022. The following keywords were entered into the PubMed database: “cleft palate,” “cleft lip,” “biofilm,” “microbiology,” “microbiome,” “bacteria,” “fungal,” and “virus.” Although no publication date limits were initially set, articles published before 1980 were not included. Reviews, systematic reviews, and meta-analyses were excluded to avoid double-counting. Only publications that were written in English and examined human subjects were included. The publications were cataloged based on the locations of the microbiota reported and the different species found between the biofilms of CL/P patients and those of non-CL/P subjects.

## 3 Results

### 3.1 Bacterial species

#### 3.1.1 Teeth inside and adjacent to the oral cleft

Given the increased prevalence of dental caries in CL/P-afflicted children (Grewcock et al., 2022), it is of particular importance to determine how the oral microbiota of those with CL/P differs from those without this condition and which bacterial species, particularly cariogenic species, may be associated with the plaque-biofilms formed on the teeth of these patients.

*Prevotella* is the most common genus differentially detected in the plaque-biofilms isolated from the teeth inside the cleft and teeth adjacent to the cleft site of CL/P patients when compared to those obtained from the teeth of healthy subjects (**Table 1** and **Supplementary Tables S1, S2**). In particular, it has been reported that *P. marshii*, *P. micans*, *P. nigrescens*, *P. pallens*, and *P. pleuritidis* are prone to CL/P, while other two species, *P. melaninogenica* and *P. oralis* (Perdikogianni et al., 2009; Funahashi et al., 2019), have been reported to be prone to a non-CL/P control group over the CL/P group (**Table 1**). Notably, there were discrepancies among associations between *Prevotella* species and the CL/P condition across publications. For example, when assessing the bacteria on all teeth, Funahashi et al. suggested that *P. loeschii* was specific to non-CL/P subjects (Funahashi et al., 2019). However, when comparing the cleft-adjacent teeth of CL/P patients with the respective incisors and canines of healthy controls, Perdikogianni et al. found no significant difference in colony counts of *P. loeschii* between the CL/P and control groups (Perdikogianni et al., 2009). A possible explanation is that the distribution of *P. loeschii* is strikingly location-specified and closely related to the cleft, which needs to be validated through further investigation.

**Table 1:** Significant differences in bacterial species obtained from the teeth of CL/P and non-CL/P patients.

Genus Frequency	Species Name	Location	Significant Difference	
			Prone to non-CL/P	Prone to CL/P
8	*Prevotella loeschii*	All teeth	Specific to non-CL/P (Funahashi et al., 2019)	
	*Prevotella marshii*	All teeth		Specific to CL/P (Funahashi et al., 2019)
	*Prevotella melaninogenica*	Inside the cleft; adjacent to the cleft	Specific to non-CL/P (Perdikogianni et al., 2009)	
	*Prevotella micans*	All teeth		Specific to CLP (Funahashi et al., 2019)
	*Prevotella nigrescens*	All teeth		Specific to CL/P (Funahashi et al., 2019)
	*Prevotella oralis*	Inside the cleft; Adjacent to the cleft	Specific to non-CL/P (Perdikogianni et al., 2009)	
	*Prevotella pallens*	All teeth		Specific to CL/P (Funahashi et al., 2019)
	*Prevotella pleuriditis*	All teeth		Specific to CL/P (Funahashi et al., 2019)
6	*Streptococcus anginosus*	All teeth		Specific to CL/P (Funahashi et al., 2019)
	*Streptococcus cristatus*	All teeth		Specific to CL/P (Funahashi et al., 2019)
	*Streptococcus gordonii*	All teeth		Specific to CL/P (Funahashi et al., 2019)
	*Streptococcus oralis*	Adjacent to the cleft	Specific to CL/P (Passinato Gheller et al., 2021)	
	*Streptococcus salivarius*	All teeth		Specific to CL/P (Funahashi et al., 2019)
	*Streptococcus sanguinis*	All teeth	Specific to non-CL/P (Funahashi et al., 2019)	
4	*Lactobacillus fermentum*	All teeth		Specific to CL/P (Funahashi et al., 2019)
	*Lactobacillus rhamnosus*	All teeth		Specific to CL/P (Funahashi et al., 2019)
	*Lactobacillus* spp.	Inside the cleft; Adjacent to the cleft		Specific to CL/P (Perdikogianni et al., 2009)
	*Lactobacillus vaginalis*	All teeth		Specific to CL/P (Funahashi et al., 2019)
2	*Aggregatibacter actinomycetemcomitans*	Adjacent to the cleft		Higher level in CL/P (Passinato Gheller et al., 2021)
	*Aggregatibacter aphrophilus*	All teeth		Specific to CL/P (Funahashi et al., 2019)
2	*Gemella hemolysans*	Inside the cleft; Adjacent to the cleft	Specific to non-CL/P (Perdikogianni et al., 2009)	
	*Gemella morbillorum*	Inside the cleft	Specific to non-CL/P (Perdikogianni et al., 2009)	
2	*Porphyromonas asaccharolyticas*	Adjacent to the cleft		Specific to CL/P (Perdikogianni et al., 2009)
	*Porphyromonas gingivalis*	Adjacent to the cleft		Specific to CL/P (Passinato Gheller et al., 2021)
1	*Anaeroglobus geminatus*	All teeth		Specific to CL/P (Funahashi et al., 2019)
1	*Bifidobacterium dentium*	All teeth		Specific to CL/P (Funahashi et al., 2019)
1	*Bilophilia wadsworthia*	Inside the cleft; Adjacent to the cleft	Specific to non-CL/P (Perdikogianni et al., 2009)	
1	*Campylobacter* spp.	Inside the cleft	Specific to non-CL/P (Perdikogianni et al., 2009)	
1	*Catonella morbi*	All teeth		Specific to CL/P (Funahashi et al., 2019)
1	*Clostridium* spp.	Inside the cleft; Adjacent to the cleft		Specific to CL/P (Perdikogianni et al., 2009)
1	*Corynebacterium matruchotii*	Inside the cleft; Adjacent to the cleft		Specific to CL/P (Perdikogianni et al., 2009)
1	*Erysipelothrix tonsillarum*	Adjacent to the cleft	Specific to non-CL/P (Funahashi et al., 2019)	
1	*Haemophilus* spp.	Adjacent to the cleft		Specific to CL/P (Perdikogianni et al., 2009)
1	*Leptotrichia buccalis*	Inside the cleft; Adjacent to the cleft		Specific to CL/P (Perdikogianni et al., 2009)
1	*Mycoplasma salivarium*	All teeth		Specific to CL/P (Funahashi et al., 2019)
1	*Mitsuokella multacida*	All teeth	Specific to non-CL/P (Funahashi et al., 2019)	
1	*Neisseria* spp.	Inside the cleft; Adjacent to the cleft		Specific to CL/P (Perdikogianni et al., 2009)
1	*Ottowia* spp.	All teeth		Specific to CL/P (Funahashi et al., 2019)
1	*Propionibacterium* spp.	Adjacent to the cleft		Specific to CL/P (Perdikogianni et al., 2009)
1	*Rothia dentocariosa*	Adjacent to the cleft		Specific to CL/P (Perdikogianni et al., 2009)
1	*Scardovia wiggsiae*	All teeth		Specific to CL/P (Funahashi et al., 2019)
1	*Selenomonas artemidis*	All teeth		Specific to CL/P (Funahashi et al., 2019)
1	*Shuttleworthia satelles*	All teeth		Specific to CL/P (Funahashi et al., 2019)
1	*Sneathia amnii*	All teeth		Specific to CL/P (Funahashi et al., 2019)
1	*Staphylococcus epidermidis*	Adjacent to the cleft		Specific to CL/P (Perdikogianni et al., 2009)
1	*Stomatobacculum longum*	All teeth	Specific to non-CL/P (Funahashi et al., 2019)	
1	*Stomatococcus* spp.	Inside the cleft; Adjacent to the cleft		Specific to CL/P (Perdikogianni et al., 2009)
1	*Tannerella forsythia*	Adjacent to cleft (supragingival)	Higher level in non-CL/P (Passinato Gheller et al., 2021)	
		Adjacent to cleft (subgingival)		Higher level in CL/P (Passinato Gheller et al., 2021)
1	*Wolinella* spp.	Inside the cleft; Adjacent to the cleft		Specific to CL/P (Perdikogianni et al., 2009)

Previous studies also demonstrate that *Streptococcus* and *Lactobacillus* are the next-most common microbiota residing on the teeth that are specific to the CL/P or non-CL/P conditions (Perdikogianni et al., 2009; Funahashi et al., 2019; Passinato Gheller et al., 2021). In regard to *Streptococcus*, only *S. anginosus*, *S. cristatus*, *S. gordonii*, and *S. salivarius* were found to be associated with CL/P, while *S. oralis* and *S. sanguinis* were associated with non-CL/P (Funahashi et al., 2019; Passinato Gheller et al., 2021). In contrast, there was no significant difference in *S. intermedius* (Perdikogianni et al., 2009; Funahashi et al., 2019), *S. mutans* (Lucas et al., 2000), and *Streptococcus* spp. prevalence (Perdikogianni et al., 2009) between these two groups. The *Lactobacillus* species found to be more prevalent in CL/P are *L. fermentum*, *L. rhamnosum*, and *L. vaginalis* (Perdikogianni et al., 2009; Funahashi et al., 2019).

Other notable pathogenic microbiota in the biofilms of teeth adjacent to the cleft associated with CL/P were *Aggregatibacter actinomycetemcomitans* (Passinato Gheller et al., 2021), *Clostridium* spp. (Perdikogianni et al., 2009), *Staphylococcus epidermidis* (Perdikogianni et al., 2009), *Tannerella forsythia* (Passinato Gheller et al., 2021), and *Neisseria* spp. (Perdikogianni et al., 2009). Interestingly, Gheller et al. (Passinato Gheller et al., 2021) found *Porphyromonas gingivalis* to be present at a higher level in both the supragingival and subgingival plaque-biofilms on teeth adjacent to the cleft in CL/P patients compared to those of the non-CL/P group (**Table 1**). However, Perdikogianni et al. (Perdikogianni et al., 2009) found no significant difference in *P. gingivalis* incidence between the CL/P and control groups when sampling subgingival plaque from the teeth in and adjacent to the cleft (**Table 1**), again suggesting the location specificity of CL/P-related oral bacteria.

#### 3.1.2 Oral, nasal, pharyngeal, and ear mucosa

Next, we further summarize the microbiota composition of mucosal surfaces. The relevant anatomic regions include the mucosa in the oral region (such as the palatal cleft site, entire palate, sublingual mucosa, and dorsum of the tongue) (**Supplementary Table S3**), and the mucosa in non-oral regions (such as the nasal mucosa, throat, oro-nasopharynx, perineum, and ear) (**Supplementary Table S4)**; in some studies, samples from both areas are combined (**Supplementary Table S5**). Although a great amount of research has been conducted on the mucosal microbiota of CL/P subjects (Mombelli et al., 1992; Machorowska-Pieniążek et al., 2017; Roode et al., 2017; Roode and Bütow, 2018; Ramdial and Madaree, 2019; Iurovschi et al., 2020), few reports compared samples from CL/P patients with those from non-CL/P subjects.

##### 3.1.2.1 The oral mucosa

*Streptococcus* is by far the most common genus identified in CL/P patients’ oral mucosa samples (**Supplementary Table S3**). Both Rodrigues et al. and Zhang et al. found *Streptococcus* in approximately one third of the CL/P patients they respectively studied (Rodrigues et al., 2021; Zhang et al., 2022). A study by Machorowska-Pieniążek et al. detected *Streptococcus* species—specifically *S. bovis* biovar I, *S. salivarius*, and *S. sanguinis*—in 20-40% of the microbiota of their CL/P patients and likewise found *S. mitis* to be highly prevalent (Machorowska-Pieniążek et al., 2017).

*Staphylococcus* is the second-most-frequent genus distributed on oral mucosa based on currently available reports (**Supplementary Table S3**). Studies examining the cleft, dorsum of the tongue (Machorowska-Pieniążek et al., 2017), and sublingual surface (Cocco et al., 2010) agreed that *S. aureus* frequently appears in these mucosal regions of CL/P patients. Machorowska-Pieniążek et al. also reported that *S. epidermidis* occurred in around a third of neonates with CL/P and more than 80% of CL/P-afflicted infants (Machorowska-Pieniążek et al., 2017); however, other studies did not find this species to occur very frequently in patients with this condition (Cocco et al., 2010).

Several other species have also been reported as frequently occurring in CL/P subjects or occurring at high levels in oral mucosa samples from these patients (**Supplementary Table S3**). For example, examination of cleft samples by Iurovschi et al. revealed that *P. melaninogenica, P. nigrescens*, and, to a lesser degree, *S. mitis* and *Enterobacter aerogenes*, had higher mean colony counts than any other species (Iurovschi et al., 2020). Multiple studies have determined *Klebsiella pneumoniae* to be moderately prevalent in CL/P patients (Cocco et al., 2010; Machorowska-Pieniążek et al., 2017). Previous research has also identified *Actinomyces viscous* to be moderately prevalent and *Gemella morbillorum* and *Veillonella* spp. to be prevalent in the oral mucosa of CL/P patients (Mombelli et al., 1992; Machorowska-Pieniążek et al., 2017). At the phylum level, Rodrigues et al. and Zhang et al. reported *Firmicutes* as occurring moderately frequently, at a greater level than any other phylum, with Zhang et al. additionally identifying *Proteobacteria* as being less prevalent (Rodrigues et al., 2021; Zhang et al., 2022).

Interestingly, Cocco et al. collected data on CL/P patients and patients with isolated cleft lip (Cocco et al., 2010), and demonstrated that although *K. pneumoniae* was separately detected in the sublingual environment and oropharynx of more than half of the CL/P patients pre-operatively, only one of the ten isolated cleft lip patients tested positive for this species in these regions (Cocco et al., 2010). Likewise, methicillin-susceptible *S. aureus* (MSSA) was identified in nearly a quarter of the CL/P patients’ sublingual specimens but was not detected in any isolated cleft lip patients, suggesting that there were substantial differences in the microbiota composition in patients with cleft lip, cleft palate, or both (Cocco et al., 2010).

##### 3.1.2.2 The mucosa of the ear, nose, and throat/pharynx

Little research has been done on the microbiota of CL/P patients’ ear mucosa. A relevant study by Chuo et al. reported that *S. aureus* occurred at a low frequency in the ear mucosa of patients with CL/P (Chuo and Timmons, 2005). In contrast, the products of ear infections, specifically otitis media secretions, have been evaluated by a greater number of studies, the results of which are discussed in Section 3.1.3.2.

Although *Staphylococcus* and *Streptococcus* are the two most-commonly detected genera occurring in the isolated nasal mucosa across the literature, only one publication (Zhang et al., 2016) has compared the prevalence of either genus in subjects with or without CL/P to date (**Supplementary Table S4**). This study by Zhang et al. found that while both genera were highly prevalent in CL/P patients, only *Streptococcus* spp. was found to be associated with the CL/P condition (Zhang et al., 2016). In comparison, other studies reported that *S. aureus* (Tuna et al., 2008), including MSSA (Cocco et al., 2010), is only moderately prevalent in CL/P patients’ nasal mucosa, with *K. pneumoniae* and *S. epidermidis* being less prevalent than this species (Cocco et al., 2010). In addition, *Corynebacterium* spp., *Dolosigranulum* spp., and *Moraxella catarrhalis* were reported as moderately prevalent, and *Gemmella* spp. and *Neisseria* spp. as less prevalent (Zhang et al., 2016). Interestingly, the genera *Bacillus* and *Dolosigranulum* were detected at a significantly lower frequency in the nasal microbiota of CL/P children than in healthy subjects (**Table 2**).

**Table 2:** Significant differences in bacterial species obtained from the nasal mucosa of CL/P and non-CL/P patients.

Genus Frequency	Species Name	Significant Difference	
		Prone to non-CL/P	Prone to CL/P
1	*Bacillus* spp.	More prevalent in non-CL/P (Zhang et al., 2016)	
1	*Dolosigranulum* spp.	More prevalent in non-CL/P (Zhang et al., 2016)	
1	*Streptococcus* spp.		More prevalent in CL/P (Zhang et al., 2016)

To date, only two studies have evaluated the bacterial microbiota of the isolated throat/oropharynx microbiota of CL/P patients; of these, Cocco et al. (Cocco et al., 2010) determined MSSA was prevalent and *K. pneumoniae* was moderately prevalent **(** **Supplementary Table S4** **)**. Interestingly, in a study by Bos et al. examining CL/P patients who tested positive for methicillin-resistant *S*. *aureus* (MRSA), 7% of subjects were positive for this bacteria in the throat only (Bos et al., 2016). A much greater proportion of patients were found to have this bacteria in both their throat mucosa and other mucosal membranes (Bos et al., 2016), indicating the importance of evaluating the microbiota of multiple regions of the mucosa.

Many studies (Chuo and Timmons, 2005; Mÿburgh and Bütow, 2009; Narinesingh et al., 2011; Thomas et al., 2012; Bos et al., 2016; Roode et al., 2017; Roode and Bütow, 2018; Ramdial and Madaree, 2019; Roode et al., 2022) have examined the pharyngeal mucosa in combination with that of the ear, nose, and the palate or palatal cleft (**Supplementary Table S5**). Two publications (Narinesingh et al., 2011; Thomas et al., 2012) evaluating nasal and/or pharyngeal samples and one publication (Chuo and Timmons, 2005) evaluating ear, nose, and throat mucosa samples reported *S. aureus* as prevalent in CL/P subjects. In contrast, a study that solely sampled the throat did not find *S. aureus* to be prevalent (Rennie et al., 2009). Two studies done by Roode et al. in 2018 and 2022 evaluating the nasopharynx and the palatal cleft or cleft palate, respectively, reported a high prevalence of *S. mitis* and *S. oralis* combined (Roode and Bütow, 2018; Roode et al., 2022). Other studies sampled from part or all of the palate and the nasopharynx or oro-nasopharynx have identified *H. influenzae*, *S. aureus*, and *S. viridans* as being prevalent and *K*. *pneumoniae* as being moderately prevalent at these sites (Mÿburgh and Bütow, 2009; Roode et al., 2017; Roode and Bütow, 2018; Ramdial and Madaree, 2019; Roode et al., 2022). Interestingly, Narinesingh et al. noticed an association between *Moraxella catarrhalis* infection and oronasal fistula formation in CL/P patients; however, this species was far from prevalent in the nasal mucosa of the study’s subjects (Narinesingh et al., 2011).

#### 3.1.3 Bodily fluids, secretions, and excretions

##### 3.1.3.1 Saliva

Saliva has multiple constituents and physicochemical properties crucial for maintaining oral health. For example, it protects the teeth and mucosa and plays an important role in maintaining balanced microbiota (Pedersen et al., 2018). While multiple studies have evaluated *S. mutans* and *Lactobacillus* in the saliva, most (Bokhout et al., 1996; de Soet et al., 1998; van Loveren et al., 1998; Arief et al., 2005; Cheng et al., 2007; Parapanisiou et al., 2009; Antoszewska et al., 2010; Cildir et al., 2012; Ritthagol et al., 2014; Sundell et al., 2015; Sundell et al., 2018; Durhan et al., 2019; Hassani et al., 2020; Chaudhari et al., 2021) have not reported a significantly greater or lesser incidence of either species in CL/P subjects compared to healthy ones (**Supplementary Table S6**). However, Shashni et al. (Shashni et al., 2015) found higher *S. mutans* counts in CL/P children with no dental caries than in non-CL/P children without caries (**Table 3**). Durhan et al. (Durhan et al., 2019) found no association between CL/P and prevalence of *S. mutans*, but reported that *Lactobacillus* was more prevalent in CL/P subjects than in healthy ones. Likewise, in 2015, Sundell et al. (Sundell et al., 2015) reported that *Lactobacillus* was more prevalent in CL/P patients than in control subjects. Surprisingly, another study conducted in 2018 by the same group (Sundell et al., 2018) found no significant difference in the occurrence of *Lactobacillus* and *S. mutans*; however, other *Streptococcus* species (including *S. gordonii*, *S. mitis*, and *S. salivarius*), *Bifidobacterium dentium*, *Fusobacterium nucleatum*, and *Veillonella parvula* were reported to be significantly less prevalent in CL/P patients **(** **Table 3** **)**. Other bacterial genera associated with the non-CL/P condition have been reported to be *Bacillus* and *Lautropia* (Zhang et al., 2016), while *S. aureus* has been found to be more prevalent in CL/P patients (Arief et al., 2005).

**Table 3:** Significant differences in bacterial species obtained from the saliva of CL/P and non-CL/P patients.

Genus Frequency	Species Name	Significant Difference	
		Prone to non-CL/P	Prone to CL/P
4	*Streptococcus gordonii*	More prevalent in non-CL/P (Sundell et al., 2018)	
	*Streptococcus mitis*	More prevalent in non-CL/P (Sundell et al., 2018)	
	*Streptococcus mutans*		Higher levels more prevalent in CL/P (Shashni et al., 2015)
	*Streptococcus salivarius*	More prevalent in non-CL/P (Sundell et al., 2018)	
1	*Bacillus* spp.	More prevalent in non-CP (Zhang et al., 2016)	
1	*Bifidobacterium dentium*	More prevalent in non-CL/P (Sundell et al., 2018)	
1	*Fusobacterium nucleatum*	More prevalent in non-CL/P (Sundell et al., 2018)	
1	*Lactobacillus* spp.		Higher levels more prevalent in CL/P (Sundell et al., 2015; Durhan et al., 2019)
1	*Lautropia* spp.	More prevalent in non-CP (Zhang et al., 2016)	
1	*Staphylococcus aureus*		More prevalent in CL/P (Arief et al., 2005)
1	*Veillonella parvula*	More prevalent in non-CL/P (Sundell et al., 2018)	

##### 3.1.3.2 Other bodily fluids, secretions, and excretions

Little research has been done to compare the bacterial compositions of the blood, feces, and otitis media secretions between CL/P and healthy populations **(** **Supplemental Table S6** **)**. However, studies solely examining CL/P patients have identified bacterial species as prevalent in these fluids. For example, Adeyemo et al. reported that coagulase-negative *Staphylococcus* was by far the most commonly-occurring bacteria in the blood of CL/P patients shortly after surgery, with over a third of the subjects testing positive for this species (Adeyemo et al., 2013). Other species, such as coagulase-positive *S. aureus*, *E. coli*, and *E. cloacae*, only occurred in a small percentage of these patients (Adeyemo et al., 2013). Meanwhile, Vieira et al. studied the presence of *Bacteroides* spp., *Bifidobacterium* spp., and *Lactobacillus* spp. in the feces of CL/P patients before and after surgical revision but did not evaluate their prevalence (Vieira et al., 2013). No doubt, further research is warranted to gain a more comprehensive understanding of the bacteria found in the blood and feces of CL/P patients compared to subjects without CL/P.

In comparison with the dearth of studies on the bacterial species in the blood and feces of CL/P patients, more research has been done on otitis media secretions **(** **Supplementary Table S6** **)**. Weckworth et al. reported that *Pseudomonas aeruginosa* was somewhat prevalent in otitis media secretions, while other species, such as *S. aureus*, *E. faecalis*, and *Proteus mirabilis*, occurred at a lower level of frequency (Weckwerth et al., 2009). A later study by the same group found *P. aeruginosa* to be the most common species and moderately prevalent, and *S. aureus* to also be prevalent (Weckwerth et al., 2014). Interestingly, when PCR analysis was utilized, the most prevalent species appeared to be *F. nucleatum*, in nearly 40% of samples (Weckwerth et al., 2014). These studies are in contrast to an earlier publication by Jousimies-Somer and Rintala, in which *M*. *catarrhalis*, *S*. *epidermidis*, and *Streptococcus pneumoniae* (but not *S*. *aureus*) were found in most CL/P infants and adolescents (Jousimies-Somer et al., 1986). While these previous investigations do contribute to current knowledge of which bacterial genera and species are prevalent in the secretions of young CL/P patients, research directly comparing CL/P patients with their healthy counterparts must be conducted to gain a more informed understanding of how the microbiota in otitis media secretions differ between these two groups.

#### 3.1.4 Bacterial species across all sites

More limited research has been done on the bacterial phyla in the tissues and fluids of CL/P patients (**Supplementary Table S7**). However, previous studies have reported *Firmicutes* to be moderately prevalent in the cleft site (Rodrigues et al., 2021) and either moderately prevalent (Zhang et al., 2022) or somewhat prevalent (Liu et al., 2016) in the saliva; *Proteobacteria* has also been found to be somewhat prevalent in the saliva (Liu et al., 2016; Zhang et al., 2022).

We also noticed that the association of certain bacterial species with the CL/P or non-CL/P condition varies among different anatomic locations. Indeed, one study may report a given species as being CL/P-specific in one location, while another might find the same species to be associated with the non-CL/P condition in another location (**Table 4**). As such, it is important to further evaluate these differences and better understand the relationship of these species with CL/P.

**Table 4:** Conflicting data on bacterial species’ association with CL/P in different locations.

Species Name	Sample Location/Tissue Type			
	Specific/Prone to non-CL/P	Specific/Prone to CL/P	No Association
*Bifidobacterium dentium*	Saliva (Sundell et al., 2018)	All teeth (Funahashi et al., 2019)	
*Bilophilia wadsworthia*	Teeth inside the cleft (Perdikogianni et al., 2009)	Teeth adjacent to the cleft (Perdikogianni et al., 2009)	
*Campylobacter* spp.	Teeth inside the cleft (Perdikogianni et al., 2009)		Teeth adjacent to the cleft (Perdikogianni et al., 2009)
*Dolosigranulum* spp.	Nose (Zhang et al., 2016)		Saliva (Zhang et al., 2016)
*Haemophilus* spp.		Teeth adjacent to the cleft (Perdikogianni et al., 2009)	Teeth inside the cleft (Perdikogianni et al., 2009)
*Lactobacillus* spp.		Teeth inside the cleft (Perdikogianni et al., 2009); Teeth adjacent to the cleft (Perdikogianni et al., 2009); Saliva (Durhan et al., 2019)	Teeth adjacent to the cleft (Lucas et al., 2000); Saliva (van Loveren et al., 1998; Cheng et al., 2007; Parapanisiou et al., 2009; Shashni et al., 2015; Chaudhari et al., 2021)
*Lautropia* spp.	Saliva (Zhang et al., 2016)		Nose (Zhang et al., 2016)
*Neisseria* spp.		Teeth inside the cleft (Perdikogianni et al., 2009); Teeth adjacent to the cleft (Perdikogianni et al., 2009)	Nose (Zhang et al., 2016); Saliva (Zhang et al., 2016)
*Porphyromonas gingivalis*		Teeth adjacent to the cleft (Perdikogianni et al., 2009; Passinato Gheller et al., 2021)	Teeth inside the cleft (Perdikogianni et al., 2009)
*Prevotella loeschii*		All teeth (Funahashi et al., 2019)	Teeth inside the cleft (Perdikogianni et al., 2009); Teeth adjacent to the cleft (Perdikogianni et al., 2009)
*Prevotella nigrescens*		Teeth adjacent to the cleft (Costa et al., 2003)	All teeth (Funahashi et al., 2019)
*Prevotella intermedia/nigrescens*			Teeth inside the cleft (Perdikogianni et al., 2009); Teeth adjacent to the cleft (Perdikogianni et al., 2009)
*Propionibacterium dentium*		Teeth adjacent to the cleft (Perdikogianni et al., 2009)	Teeth inside the cleft (Perdikogianni et al., 2009)
*Rothia dentocariosa*		Teeth adjacent to the cleft (Perdikogianni et al., 2009)	Teeth inside the cleft (Perdikogianni et al., 2009); Saliva (Sundell et al., 2018)
*Staphylococcus epidermidis*		Teeth adjacent to the cleft (Perdikogianni et al., 2009)	Teeth inside the cleft (Perdikogianni et al., 2009)
*Streptococcus gordonii*	Saliva (Sundell et al., 2018)	All teeth (Funahashi et al., 2019)	
*Streptococcus mutans*		Saliva (Shashni et al., 2015; Sundell et al., 2015)	Teeth adjacent to the cleft (Lucas et al., 2000); Saliva (van Loveren et al., 1998; Cheng et al., 2007; Parapanisiou et al., 2009; Antoszewska et al., 2010; Sundell et al., 2018; Durhan et al., 2019; Chaudhari et al., 2021)
*Streptococcus salivarius*	Saliva (Sundell et al., 2018)	All teeth (Funahashi et al., 2019)	
*Streptococcus* spp.		Nose (Zhang et al., 2016)	Teeth inside the cleft (Perdikogianni et al., 2009); Teeth adjacent to the cleft (Perdikogianni et al., 2009); Saliva (Zhang et al., 2016)
*Tannerella forsythia*	Teeth adjacent to the cleft (supragingival) (Passinato Gheller et al., 2021)	Teeth adjacent to the cleft (subgingival) (Passinato Gheller et al., 2021)	
*Veillonella parvula*	Saliva (Sundell et al., 2018)	All teeth (Funahashi et al., 2019)	

### 3.2 Fungi

*Candida* species typically form colonies in the mouth, digestive tract, skin, and vagina; excessive growth of these colonies may result in fungal infections that can lead to hospitalization or even death (Singh et al., 2020). *Candida albicans* is particularly pathogenic and has great capability for forming biofilms (Poulain, 2015; Ponde et al., 2021). Thus, examining the prevalence of *C. albicans* in the oral flora of CL/P patients is critical for understanding the nontypical nature of CL/P oral biofilms (**Table 5**).

**Table 5:** Significant differences in *Candida* spp. and *Candida* species in CL/P and non-CL/P patients.

Genus Frequency	Species Name	Location	Significant Difference	
			Prone to non-CL/P	Prone to CL/P
3	*Candida* spp.	Sublingual region, tongue dorsum, palate, buccal mucosa, and gingival margin (de Souza et al., 2022); Oral rinse (Boriollo et al., 2022); Tongue dorsum and buccal and palatal mucosae (Rawashdeh et al., 2011)		More prevalent in CL/P (Rawashdeh et al., 2011; Boriollo et al., 2022; de Souza et al., 2022)

Previous research sampling various oral mucosae has reported *Candida* spp. as being highly prevalent in CL/P patients (Rawashdeh et al., 2011; Boriollo et al., 2022; de Souza et al., 2022), although this association was disputed in a study by Durhan et al., in which the saliva was evaluated (Durhan et al., 2019). Other studies also reported this fungus as being moderately prevalent (Durhan et al., 2019), somewhat prevalent (Silva et al., 2018), and not prevalent in CL/P patients (Yilmaz et al., 2020). It is worth noting that although *C. albicans* is by far the most commonly-investigated fungal species in studies examining the oral environment of CL/P patients, the prevalence of *C. albicans* in CL/P patients varies across studies, ranging from less than 10% to as high as 70% (Mattos et al., 2009; Rawashdeh et al., 2011; Machorowska-Pieniążek et al., 2017; Yilmaz et al., 2020; Boriollo et al., 2022). Interestingly, Boriollo et al. reported that colonization by non-*albicans Candida* species only (namely, *Candida krusei* and *Candida tropicalis*) was more prevalent in CL/P patients than in the control (Boriollo et al., 2022). On the contrary, Rawashdeh et al. (Rawashdeh et al., 2011) reported that *C. kefyr* was non-significantly more prevalent in healthy subjects who tested positive for *Candida* species compared to their CL/P counterparts (**Supplementary Table S8**).

The prevalence of different *Candida* species in the same location also varies. Silva et al. detected *Candida* spp. in the cleft site in 40% of CL/P patients, while the prevalence of *C. albicans* in the same site was only around 15% (Silva et al., 2018). In comparison, other *Candida* species, such as *C. krusei* and *C. tropicalis*, were found at even lower levels in the cleft (Silva et al., 2018). Similarly, Roode and Bütow reported that nearly a third of the CL/P subjects harbored *C. albicans* at the soft palate margin and the nasopharyngeal mucosa, while *C. tropicalis* and *C. krusei* were much less prevalent at these locations (Roode and Bütow, 2018).

### 3.3 Viruses

The association between viral infection and CL/P has also been evaluated (Cerný et al., 1991; Acs et al., 2005; Métneki et al., 2005; Divya et al., 2017; Ács et al., 2020); however, there is no available data to demonstrate if any viruses participate in CL/P-specific oral or nasal biofilm formation (**Supplementary Table S9**).

## 4 Discussion

**Figure 1:**
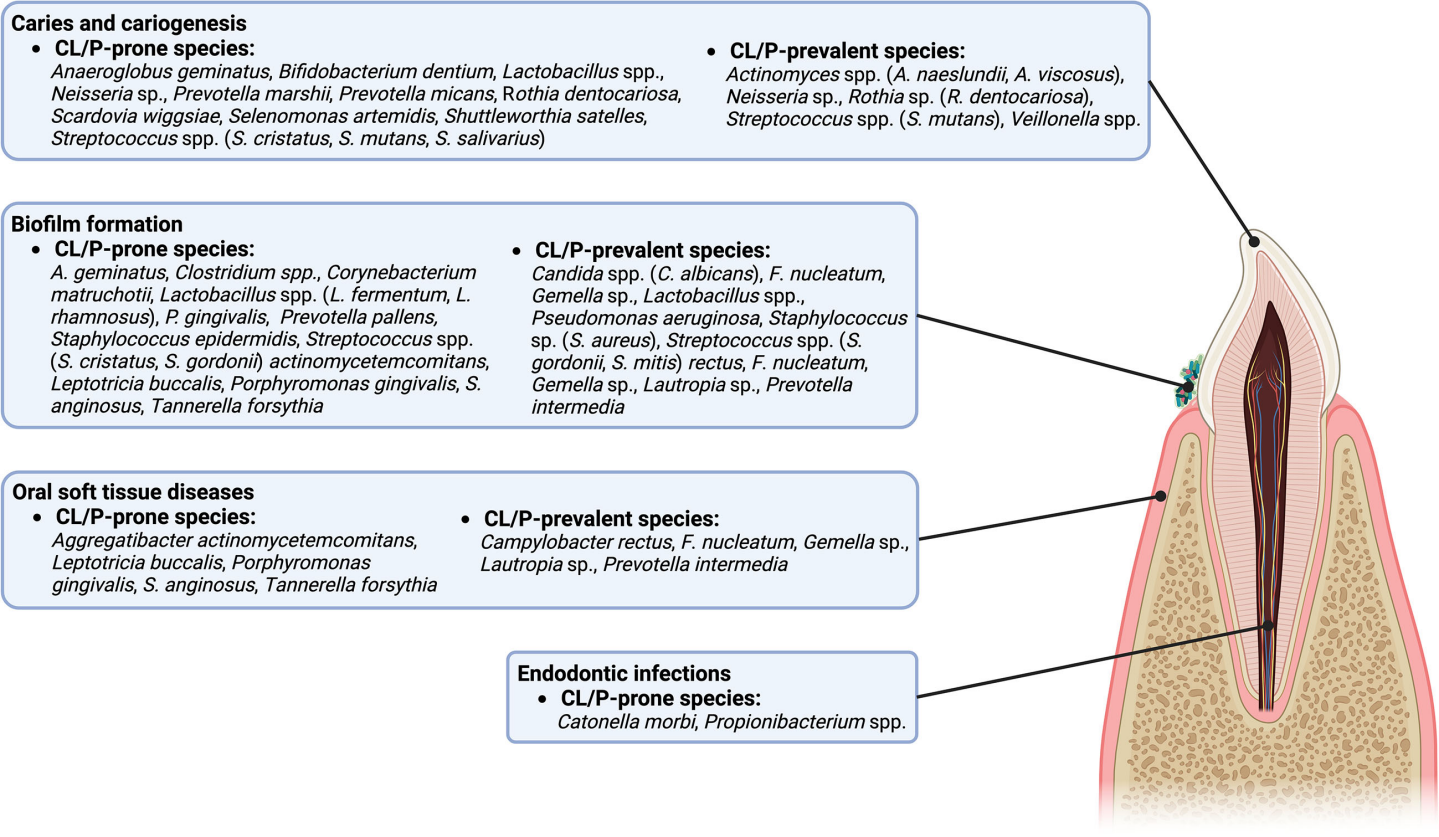
A diagram of the intraoral anatomic structures that are potentially influenced by CL/P-prone and CL/P-prevalent species.

The current review summarized the significant differences between the microbiota of those with and without CL/P. Many CL/P-prone microbes, whether specific to CL/P patients or more prevalent in CL/P patients than in their healthy counterparts, have been implicated in oral health and disease (**Figure 1**). For example, there are a multitude of CL/P-prone microbes associated with caries, including *Bifidobacterium dentium*, *Lactobacillus* spp., a *Neisseria* sp., *Rothia dentocariosa*, *Scardovia wiggsiae*, *Streptococcus cristatus*, *Streptococcus mutans*, and *Streptococcus salivarius* (Kressirer et al., 2017; Inquimbert et al., 2019; Xiao et al., 2021), while *Anaeroglobus geminatus* and *Shuttleworthia satelles* have been found to be more prevalent in patients with caries (Wang et al., 2019). Indeed, several CL/P-prone microbes, such as *P. marshii*, *P. micans*, *S. wiggsiae, Selenomonas artemidis*, and *S. satelles*, produce acidic byproducts from sugar metabolism, contributing to the development of an acidic oral environment, and, potentially, caries (Moore et al., 1987; Downes et al., 2002; Downes et al., 2005; Downes et al., 2009; Kressirer et al., 2017). Moreover, *Catonella morbi* has been implicated in a more serious affliction of the teeth, primary endodontic infections, while *Propionibacterium* has been implicated in secondary endodontic infections and lesions (Siqueira and Rôças, 2006; Dioguardi et al., 2020).

CL/P-prone microbes have also been associated with oral inflammatory diseases and soft tissue destruction (**Figure 1**). For example, a link between gingivitis and the CL/P-prone bacteria *T*. *forsythia* (Sharma, 2010) has been reported. Meanwhile, associations between species such as *A*. *actinomycetemcomitans*, *P. gingivalis*, *S*. *anginosus*, and *T. forsythia* and periodontitis have been reported (Socransky et al., 1998; Kumagai et al., 2003; Raja et al., 2014; How et al., 2016). It is possible that *L*. *buccalis* may also be associated with one or more maladies of the oral soft tissue (Eribe and Olsen, 2017).

The CL/P-prone microbes that contribute to biofilm formation and development are particularly interesting (**Figure 1**). Several bacteria, such as *Clostridium*, *Corynebacterium matruchotii*, *P*. *gingivalis*, *S. epidermidis*, and *S. gordonii* are known to form biofilms or promote biofilm formation on tooth surfaces (Otto, 2009; Pantaléon et al., 2014; Gerits et al., 2017; Rath et al., 2017; Esberg et al., 2020), while *P. pallens* is recognized as an early colonizer (Könönen and Gursoy, 2021). *Candida* spp. colonization also promotes the formation of biofilms (Cavalheiro and Teixeira, 2018), potentially affecting the oral health through the development of thrush (also known as pseudomembranous candidiasis) (Akpan and Morgan, 2002). Interestingly, some microbes work in conjunction with others to enhance the formation of oral biofilms. For example, *A. geminatus* promotes the growth of *P. intermedia* (Bao et al., 2017). In contrast, some species act as antagonists, limiting the ability of other microbes to reside or accumulate on oral surfaces (Wang et al., 2009; Melo et al., 2016; Ho et al., 2017; Tahmourespour et al., 2019; He et al., 2021). Examples include CL/P-prone *Lactobacillus* species such as *L. fermentum*, which can inhibit *S. aureus* (Melo et al., 2016); and *L. rhamnosus*, which can inhibit *Gardnerella* (He et al., 2021) and *S. mutans* (Tahmourespour et al., 2019). Other antagonists include *S. cristatus*, which can restrict *P. gingivalis* colonization and virulence (Ho et al., 2017), and *A. geminatus*, which downregulates multiple proteins produced by *S. oralis* (Wang et al., 2009; Bao et al., 2017). Overall, microbiota prone to CL/P contribute to a specific microbe community which may lead to CL/P-specific infectious complications or worsen the prognosis of infections.

**Figure 2:**
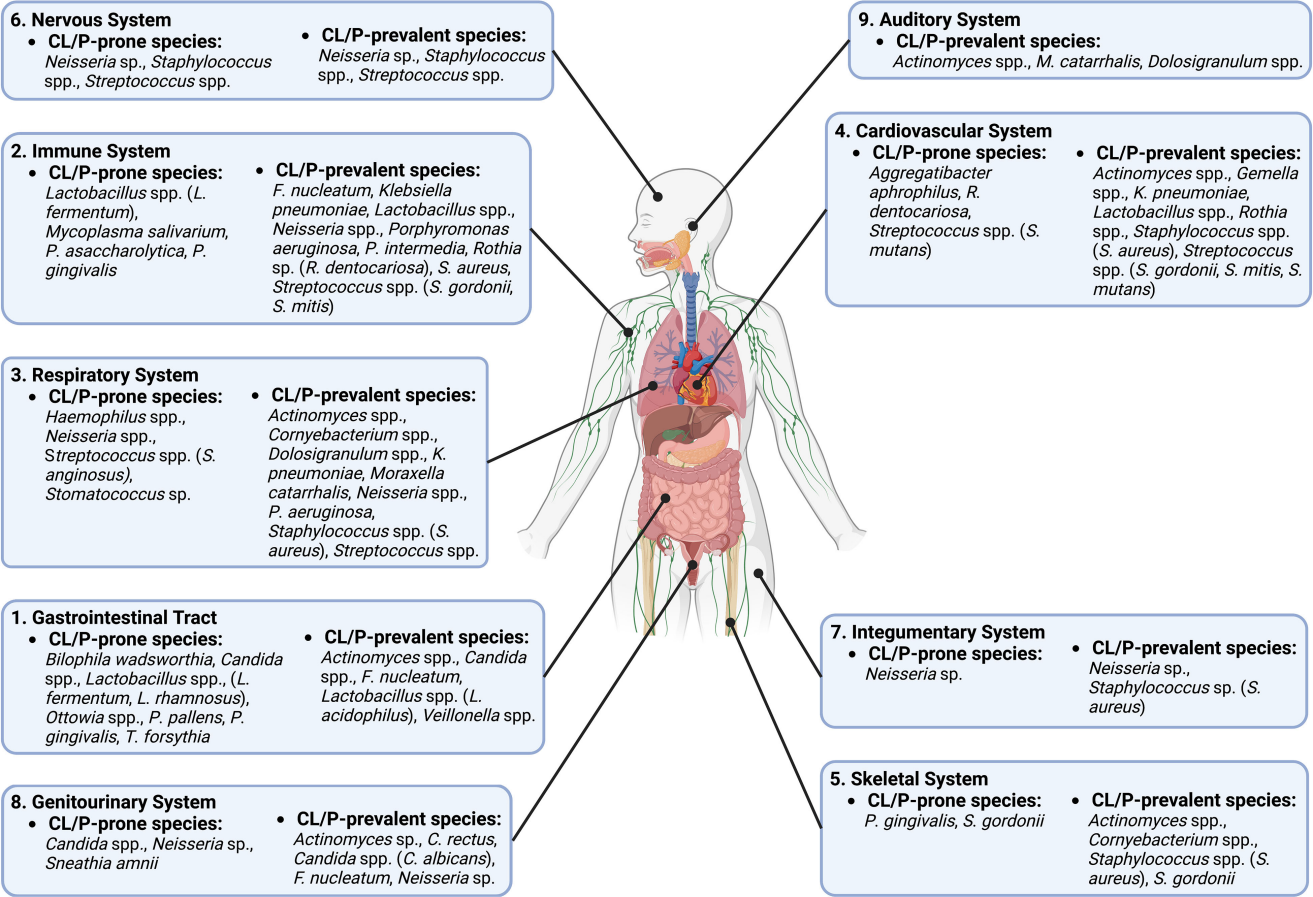
A diagram of different body systems that are potentially influenced by CL/P-prone and CL/P-prevalent species.

Besides the oral cavity, CL/P-prone microbes also affect other parts of the digestive tract (**Figure 2**). For example, *L. fermentum* and *L. rhamnosus* may be associated with oropharyngeal cancer (Guerrero-Preston et al., 2017); *P*. *gingivalis* and *T*. *forsythia* have been linked to esophageal cancer (Malinowski et al., 2019); *Ottowia* is found in higher abundance in patients with Crohn’s Disease (Sun et al., 2021); *Bilophila wadsworthia* can cause gastrointestinal (GI) tract inflammation and disrupt the functioning of the intestinal barrier in those who consume foods high in fat (Natividad et al., 2018) and may contribute to the initiation of colorectal cancer due to its hydrogen sulfide-releasing capabilities (Phipps et al., 2020); and *P*. *pallens* is less abundant in the saliva of those with reflux (Kawar et al., 2021), with there also being an inverse relationship between the severity of some gastritis symptoms and abundance of *P. pallens* (Han et al., 2019). In addition, *Candida* spp. can cause candidiasis in the GI tract (Sprague et al., 2022).

Particular CL/P-prone microbes also influence the immune system (**Figure 2**). For example, *P. gingivalis*, which has been reported to modulate inflammation in a variety of tissues (How et al., 2016; Chen et al., 2018; Bugueno et al., 2020), has an inhibitory effect on communication between Toll-like receptors and destroys cytokines produced by the host’s cells (Hajishengallis and Diaz, 2020). *Mycoplasma salivarium* has been shown to disrupt monocyte and macrophage activity, including phagocytosis (Nolan et al., 2016). *Lactobacillus* spp. likewise modulate immune cells: one particular strain of *L. fermentum* can upregulate specific interleukins in T-lymphocytes, while another strain has the same effect on dendritic cells (Ding et al., 2017). *L. fermentum* can also reduce intestinal inflammation (Aoudia et al., 2016), while *P*. *asaccharolytica* is known to boost the release of interleukins and tumor necrosis factors (Magalashvili et al., 2008).

CL/P-prone microbes affect myriad other systems of the body (**Figure 2**). For example, studies indicate that *S. anginosus*, a *Haemophilus* sp., a *Neisseria* sp., and a *Stomatococcus* sp. promote infection and disease of the respiratory system (King, 2012; Deutschmann et al., 2013; Yuan et al., 2013; Noguchi et al., 2015; Li et al., 2022). The circulatory system is also affected by CL/P-prone microbes: *R. dentocariosa* biofilm formation in the heart can promote ineffective endocarditis (Greve et al., 2021), a condition that is also stimulated by *S. mutans* and *A. aphrophilus* (Nørskov-Lauritsen, 2014; Nomura et al., 2020); *S. mutans* has also been linked to the development of atherosclerotic plaque (Kesavalu et al., 2012). In addition, both *P*. *gingivalis* and *S. gordonii* can cause bone resorption (Kassem et al., 2015; Park et al., 2020), while *Streptococcus* spp. colonization may be linked to brain abscesses and meningitis (Bokhari and Mesfin, 2023; Mandziuk and Kuchar, 2023). Previously studies also showed that a *Neisseria* sp. can cause dermatitis (da Cruz et al., 2019) and is the cause of the sexually-transmitted disease gonorrhea, which can cause serious harm to the genitourinary system (Unemo et al., 2019). Meanwhile, another CLP-prone bacterial species, *Sneathia amnii*, is associated with cervical cancer and appears to be capable of binding to and having a cytotoxic effect on cervical cancer cells (Nawrot et al., 2010; Harwich et al., 2012). Moreover, *S*. *amnii* is of great concern in reproduction, as this bacterial species produces an exotoxin that permeabilizes fetal membrane cells and is associated with stillbirths and conditions such as preeclampsia that may jeopardize the lives of the mother and unborn child (Harwich et al., 2012; Vitorino et al., 2019; Gentile et al., 2020).

On the other hand, multiple microbe species that have been found to be more prevalent in non-CL/P subjects than in CL/P patients may have a positive effect on human health. For example, certain species of *Dolosigranulum* may be negatively correlated with the incidence of particular respiratory maladies and enhance the immune response of the respiratory epithelium (Islam et al., 2021; Nesbitt et al., 2021) and appear to provide resistance to ear infections (Pettigrew et al., 2012; Lappan et al., 2018)—a common complaint of CL/P patients (Flynn et al., 2009). Strikingly, lack of *Mitsuokella multacida*, another species specific to the non-CL/P condition, is associated with the early development of colon cancer (Arafat, 2020; Elkholy et al., 2020), although its presence may be linked to squamous cell lung cancer as well (Zhao et al., 2021). Undoubtedly, further research is necessary to determine if and how these microbes affect the health of normal and CL/P populations.

## 5 Conclusion

Significant differences in the prevalence of particular microbiota have been found in various anatomic locations of CL/P patients in comparison with those of non-CL/P subjects. Many such species have deleterious effects on specific tissues or are associated with serious diseases, making it imperative to definitively investigate the microbiota of CL/P patients to have a more complete understanding of their condition. Interestingly, characterization of a given species as CL/P-prone or non-CL/P-prone was not consistent among different tissue sampling sites, indicating that further research must be done to fully understand the non-typical bacterial and fungal species particular to CL/P patients.

## Author contributions

ZZ and CL contributed to conception and design of the study. EG, BX, and MS performed the article searching and data organization. EG, YL, and BX wrote the first draft of the manuscript. EG and BX drafted the figures. HK, ZZ, and CL did critical revision of the manuscript. All authors contributed to the article and approved the submitted version.
