# Supplementary material for: Characterizing the microbiota of cleft lip and palate patients: a comprehensive review
Source: Front Cell Infect Microbiol. 2023 Apr 18;13:1159455. doi: 10.3389/fcimb.2023.1159455 (PMC10152472; doi:10.3389/fcimb.2023.1159455)
Supplement: Supplementary file 1 [file Table_1.docx]

Supplementary Material

Characterizing the Microbiota of Cleft Lip and Palate Patients: A Comprehensive Review

Elizabeth Gershater, Yuan Liu, Binglan Xue, Min Kyung Shin, Hyun Koo, Zhong Zheng^*^, Chenshuang Li^*^

*** Correspondence:** Zhong Zheng: leozz95@gmail.com; Chenshuang Li: lichens@upenn.edu

**Supplementary Table S1. Bacteria identified on teeth inside the oral cleft of CL/P and non-CL/P patients.**

| **Genus Frequency** | **Species Name** | **Prevalence in CL/P** | **Significant Difference** | | |
| --- | --- | --- | --- | --- | --- |
|  |  |  | **Prone to non-CL/P** | **Prone to CL/P** | **Not Prone to Either** |
| 5 | *Prevotella intermedia* | Moderately prevalent (Quirynen et al., 2003) | N/A (Quirynen et al., 2003) | | |
|  | *Prevotella intermedia/nigrescens* | Not prevalent (Perdikogianni et al., 2009) |  |  | (Perdikogianni et al., 2009) |
|  | *Prevotella loeschii* | Not prevalent (Perdikogianni et al., 2009) |  |  | (Perdikogianni et al., 2009) |
|  | *Prevotella melaninogenica* | Not prevalent (Perdikogianni et al., 2009) | (Perdikogianni et al., 2009) |  |  |
|  | *Prevotella oralis* | Not prevalent (Perdikogianni et al., 2009) | (Perdikogianni et al., 2009) |  |  |
| 2 | *Streptococcus intermedius* | Not prevalent (Perdikogianni et al., 2009) |  |  | (Perdikogianni et al., 2009) |
|  | *Streptococcus* spp. | Not prevalent (Perdikogianni et al., 2009) |  |  | (Perdikogianni et al., 2009) |
| 2 | *Actinomyces israelii* | Not prevalent (Perdikogianni et al., 2009) |  |  | (Perdikogianni et al., 2009) |
|  | *Actinomyces* spp. | Not prevalent (Perdikogianni et al., 2009) |  |  | (Perdikogianni et al., 2009) |
| 2 | *Campylobacter rectus* | Moderately prevalent (Quirynen et al., 2003) | N/A (Quirynen et al., 2003) | | |
|  | *Campylobacter* spp. | Not prevalent (Perdikogianni et al., 2009) | (Perdikogianni et al., 2009) |  |  |
| 2 | *Fusobacterium nucleatum* | Highly prevalent (Quirynen et al., 2003) | N/A (Quirynen et al., 2003) | | |
|  | *Fusobacterium* spp. | Not prevalent (Perdikogianni et al., 2009) |  |  | (Perdikogianni et al., 2009) |
| 2 | *Gemella hemolysans* | Not prevalent (Perdikogianni et al., 2009) | (Perdikogianni et al., 2009) |  |  |
|  | *Gemella morbillorum* | Not prevalent (Perdikogianni et al., 2009) | (Perdikogianni et al., 2009) |  |  |
| 2 | *Porphyromonas assaccharolytica* | Not prevalent (Perdikogianni et al., 2009) |  |  | (Perdikogianni et al., 2009) |
|  | *Porphyromonas gingivalis* | Not prevalent (Perdikogianni et al., 2009) |  |  | (Perdikogianni et al., 2009) |
| 1 | *Bacteroides* spp. | Not prevalent (Perdikogianni et al., 2009) |  |  | (Perdikogianni et al., 2009) |
| 1 | *Bifidobacterium* spp. | Not prevalent (Perdikogianni et al., 2009) |  |  | (Perdikogianni et al., 2009) |
| 1 | *Bilophila wadsworthia* | Not prevalent (Perdikogianni et al., 2009) | (Perdikogianni et al., 2009) |  |  |
| 1 | *Capnocytophaga* spp. | Not prevalent (Perdikogianni et al., 2009) |  |  | (Perdikogianni et al., 2009) |
| 1 | *Clostridium* spp. | Not prevalent (Perdikogianni et al., 2009) |  | (Perdikogianni et al., 2009) |  |
| 1 | *Corynebacterium matruchotii* | Not prevalent (Perdikogianni et al., 2009) |  | (Perdikogianni et al., 2009) |  |
| 1 | *Eikenella corrodens* | Not prevalent (Perdikogianni et al., 2009) |  |  | (Perdikogianni et al., 2009) |
| 1 | *Eubacterium* spp. | Not prevalent (Perdikogianni et al., 2009) |  |  | (Perdikogianni et al., 2009) |
| 1 | *Haemophilus* spp. | Not prevalent (Perdikogianni et al., 2009) |  |  | (Perdikogianni et al., 2009) |
| 1 | *Lactobacillus* spp. | Not prevalent (Perdikogianni et al., 2009) |  | (Perdikogianni et al., 2009) |  |
| 1 | *Leptotrichia buccalis* | Not prevalent (Perdikogianni et al., 2009) |  | (Perdikogianni et al., 2009) |  |
| 1 | *Neisseria* spp. | Not prevalent (Perdikogianni et al., 2009) |  | (Perdikogianni et al., 2009) |  |
| 1 | *Parvimonas micra* | Not prevalent (Perdikogianni et al., 2009) |  |  | (Perdikogianni et al., 2009) |
| 1 | *Peptostreptococcus micra* | Somewhat prevalent (Quirynen et al., 2003) | N/A (Quirynen et al., 2003) | | |
| 1 | *Propionibacterium* spp. | Not prevalent (Perdikogianni et al., 2009) |  |  | (Perdikogianni et al., 2009) |
| 1 | *Rothia dentocariosa* | Not prevalent (Perdikogianni et al., 2009) |  |  | (Perdikogianni et al., 2009) |
| 1 | *Selenomonas* spp. | Not prevalent (Perdikogianni et al., 2009) |  |  | (Perdikogianni et al., 2009) |
| 1 | *Staphylococcus epidermidis* | Not prevalent (Perdikogianni et al., 2009) |  |  | (Perdikogianni et al., 2009) |
| 1 | *Stomatococcus* spp. | Not prevalent (Perdikogianni et al., 2009) |  | (Perdikogianni et al., 2009) |  |
| 1 | *Veillonella* spp. | Not prevalent (Perdikogianni et al., 2009) |  |  | (Perdikogianni et al., 2009) |
| 1 | *Wolinella* spp. | Not prevalent (Perdikogianni et al., 2009) |  | (Perdikogianni et al., 2009) |  |

**Supplementary Table S2. Bacteria identified on teeth adjacent to the cleft* in CL/P and non-CL/P patients. *For Funahashi *et al*., all teeth were sampled.**

| **Genus Frequency** | **Species Name** | **Prevalence in CL/P** | **Significant Difference** | | | | | |
| --- | --- | --- | --- | --- | --- | --- | --- | --- |
|  |  |  | **Prone to non-CL/P** | | **Prone to CL/P** | | | **Not Prone to Either** |
| 27 | *Streptococcus anginosus* | Not prevalent (Funahashi et al., 2019); N/A (Iurovschi et al., 2020) |  | | (Funahashi et al., 2019) | | |  |
|  |  |  | N/A (Iurovschi et al., 2020) | | | | | |
|  | *Streptococcus constellatus* | N/A (Iurovschi et al., 2020) | N/A (Iurovschi et al., 2020) | | | | | |
|  | *Streptococcus cristatus* | Not prevalent (Funahashi et al., 2019) |  | | (Funahashi et al., 2019) | | |  |
|  | *Streptococcus gordonii* | Not prevalent (Funahashi et al., 2019); N/A (Iurovschi et al., 2020) |  | | (Funahashi et al., 2019) | | |  |
|  |  |  | N/A (Iurovschi et al., 2020) | | | | | |
|  | *Streptococcus intermedius* | Not prevalent (Perdikogianni et al., 2009; Funahashi et al., 2019); N/A (Iurovschi et al., 2020) |  | |  | | | (Perdikogianni et al., 2009; Funahashi et al., 2019) |
|  |  |  | N/A (Iurovschi et al., 2020) | | | | | |
|  | *Streptococcus mitis* | Highly prevalent (Machorowska-Pieniążek et al., 2013); N/A (Iurovschi et al., 2020) | N/A (Machorowska-Pieniążek et al., 2013; Iurovschi et al., 2020) | | | | | |
|  | *Streptococcus mutans* | Moderately prevalent to highly prevalent (Lucas et al., 2000); Moderately prevalent (Bokhout et al., 1996; van Loveren et al., 1998); N/A (Iurovschi et al., 2020) |  | |  | | | (Lucas et al., 2000) |
|  |  |  | N/A (Bokhout et al., 1996; van Loveren et al., 1998; Iurovschi et al., 2020) | | | | | |
|  | *Streptococcus oralis* | Not prevalent (Machorowska-Pieniążek et al., 2013); N/A (Iurovschi et al., 2020; Passinato Gheller et al., 2021) | (Passinato Gheller et al., 2021) | |  | | |  |
|  |  |  | N/A (Machorowska-Pieniążek et al., 2013; Iurovschi et al., 2020) | | | | | |
|  | *Streptococcus pyogenes* | N/A (Iurovschi et al., 2020) | N/A (Iurovschi et al., 2020) | | | | | |
|  | *Streptococcus salivarius* | Somewhat prevalent (Machorowska-Pieniążek et al., 2013); Not prevalent (Funahashi et al., 2019) |  | | (Funahashi et al., 2019) | | |  |
|  |  |  | N/A (Machorowska-Pieniążek et al., 2013) | | | | | |
|  | *Streptococcus sanguinis* | Somewhat prevalent (Machorowska-Pieniążek et al., 2013); Not prevalent (Funahashi et al., 2019); N/A (Iurovschi et al., 2020) | (Funahashi et al., 2019) | |  | | |  |
|  |  |  | N/A (Machorowska-Pieniążek et al., 2013; Iurovschi et al., 2020) | | | | | |
|  | *Streptococcus* spp. | Highly prevalent (Machorowska-Pieniążek et al., 2013); Somewhat prevalent (Perdikogianni et al., 2009) |  | |  | | | (Perdikogianni et al., 2009) |
|  |  |  | N/A (Machorowska-Pieniążek et al., 2013) | | | | | |
|  | *Streptococcus vestibularis* | Somewhat prevalent (Machorowska-Pieniążek et al., 2013) | N/A (Machorowska-Pieniążek et al., 2013) | | | | | |
| 22 | *Prevotella assaccharolytica* | Not prevalent (Funahashi et al., 2019) |  |  | | | (Funahashi et al., 2019) | |
|  | *Prevotella intermedia* | Moderately prevalent (Mombelli et al., 1992; Quirynen et al., 2003); N/A (Iurovschi et al., 2020) | N/A (Mombelli et al., 1992; Quirynen et al., 2003; Iurovschi et al., 2020) | | | | | |
|  | *Prevotella intermedia/nigrescens* | Not prevalent (Perdikogianni et al., 2009) |  | |  | | | (Perdikogianni et al., 2009) |
|  | *Prevotella loeschii* | Not prevalent (Perdikogianni et al., 2009; Funahashi et al., 2019) | (Funahashi et al., 2019) | |  | | | (Perdikogianni et al., 2009) |
|  | *Prevotella maculosa* | Not prevalent (Funahashi et al., 2019) |  | |  | | | (Funahashi et al., 2019) |
|  | *Prevotella marshii* | Not prevalent (Funahashi et al., 2019) |  | | (Funahashi et al., 2019) | | |  |
|  | *Prevotella melaninogenica* | Not prevalent (Mombelli et al., 1992; Perdikogianni et al., 2009); N/A, average count higher than other species (Iurovschi et al., 2020) | (Perdikogianni et al., 2009) | |  | | |  |
|  |  |  | N/A (Mombelli et al., 1992; Iurovschi et al., 2020) | | | | | |
|  | *Prevotella melaninogenica*/*loeschi*/*denticola* | Not prevalent (Mombelli et al., 1992) | N/A (Mombelli et al., 1992) | | | | | |
|  | *Prevotella micans* | Not prevalent (Funahashi et al., 2019) |  | | (Funahashi et al., 2019) | | |  |
|  | *Prevotella nigrescens* | Not prevalent (Costa et al., 2003; Funahashi et al., 2019); N/A, average count higher than other species (Iurovschi et al., 2020) |  | | (Funahashi et al., 2019) | | | (Costa et al., 2003) |
|  |  |  | N/A (Iurovschi et al., 2020) | | | | | |
|  | *Prevotella oralis* | Not prevalent (Perdikogianni et al., 2009) | (Perdikogianni et al., 2009) | |  | | |  |
|  | *Prevotella oris* | Not prevalent (Funahashi et al., 2019) |  | |  | | | (Funahashi et al., 2019) |
|  | *Prevotella pallens* | Not prevalent (Funahashi et al., 2019) |  | | (Funahashi et al., 2019) | | |  |
|  | *Prevotella pleuritidis* | Not prevalent (Funahashi et al., 2019) |  | | (Funahashi et al., 2019) | | |  |
|  | *Prevotella veroralis* | Not prevalent (Funahashi et al., 2019) |  | |  | | | (Funahashi et al., 2019) |
| 13 | *Actinomyces gerencseriae* | N/A (Iurovschi et al., 2020) | N/A (Iurovschi et al., 2020) | | | | | |
|  | *Actinomyces israelii* | Somewhat prevalent (Machorowska-Pieniążek et al., 2013); Not prevalent (Perdikogianni et al., 2009); N/A (Iurovschi et al., 2020) |  | |  | | | (Perdikogianni et al., 2009) |
|  |  |  | N/A (Machorowska-Pieniążek et al., 2013; Iurovschi et al., 2020) | | | | | |
|  | *Actinomyces johnsonii* | Not prevalent (Funahashi et al., 2019) |  | |  | | | (Funahashi et al., 2019) |
|  | *Actinomyces meyeri* | N/A (Iurovschi et al., 2020) | N/A (Iurovschi et al., 2020) | | | | | |
|  | *Actinomyces naeslundii* | N/A (Iurovschi et al., 2020) | N/A (Iurovschi et al., 2020) | | | | | |
|  | *Actinomyces odontolyticus* | Not prevalent (Funahashi et al., 2019); N/A (Iurovschi et al., 2020) |  | |  | | | (Funahashi et al., 2019) |
|  |  |  | N/A (Iurovschi et al., 2020) | | | | | |
|  | *Actinomyces oris* | N/A (Iurovschi et al., 2020) | N/A (Iurovschi et al., 2020) | | | | | |
|  | *Actinomyces* spp. | Moderately prevalent (Machorowska-Pieniążek et al., 2013); Not prevalent (Perdikogianni et al., 2009) |  | |  | | | (Perdikogianni et al., 2009) |
|  |  |  | N/A (Machorowska-Pieniążek et al., 2013) | | | | | |
|  | *Actinomyces viscosus* | Moderately prevalent (Mombelli et al., 1992) | N/A (Mombelli et al., 1992) | | | | | |
| 9 | *Fusobacterium necrophorum* | N/A (Iurovschi et al., 2020) | N/A (Iurovschi et al., 2020) | | | | | |
|  | *Fusobacterium nucleatum* | Highly prevalent (Quirynen et al., 2003) | N/A (Quirynen et al., 2003) | | | | | |
|  | *Fusobacterium nucleatum nucleatum* | N/A (Iurovschi et al., 2020) | N/A (Iurovschi et al., 2020) | | | | | |
|  | *Fusobacterium nucleatum polymorphum* | N/A (Iurovschi et al., 2020) | N/A (Iurovschi et al., 2020) | | | | | |
|  | *Fusobacterium nucleatum vincentii* | N/A (Iurovschi et al., 2020) | N/A (Iurovschi et al., 2020) | | | | | |
|  | *Fusobacterium periodonticum* | N/A (Iurovschi et al., 2020) | N/A (Iurovschi et al., 2020) | | | | | |
|  | *Fusobacterium* spp. | Somewhat prevalent (Mombelli et al., 1992; Machorowska-Pieniążek et al., 2013); Not prevalent (Perdikogianni et al., 2009) |  | |  | | | (Perdikogianni et al., 2009) |
|  |  |  | N/A (Mombelli et al., 1992; Machorowska-Pieniążek et al., 2013) | | | | | |
| 9 | *Lactobacillus acidophilus* | N/A (Iurovschi et al., 2020) | N/A (Iurovschi et al., 2020) | | | | | |
|  | *Lactobacillus fermentum* | Not prevalent (Funahashi et al., 2019); N/A (Iurovschi et al., 2020) |  | | (Funahashi et al., 2019) | | |  |
|  |  |  | N/A (Iurovschi et al., 2020) | | | | | |
|  | *Lactobacillus rhamnosus* | Not prevalent (Funahashi et al., 2019) |  | | (Funahashi et al., 2019) | | |  |
|  | *Lactobacillus* spp. | Not prevalent to somewhat prevalent (Lucas et al., 2000); Not prevalent (Bokhout et al., 1996; van Loveren et al., 1998; Perdikogianni et al., 2009) |  | | (Perdikogianni et al., 2009) | | | (Lucas et al., 2000) |
|  |  |  | N/A (Bokhout et al., 1996; van Loveren et al., 1998) | | | | | |
|  | *Lactobacillus vaginalis* | Not prevalent (Funahashi et al., 2019) |  | | (Funahashi et al., 2019) | | |  |
| 7 | *Capnocytophaga gingivalis* | Not prevalent (Funahashi et al., 2019); N/A (Iurovschi et al., 2020) |  | | (Funahashi et al., 2019) | | |  |
|  |  |  | N/A (Iurovschi et al., 2020) | | | | | |
|  | *Capnocytophaga ochracea* | N/A (Iurovschi et al., 2020) | N/A (Iurovschi et al., 2020) | | | | | |
|  | *Capnocytophaga* spp. | Somewhat prevalent (Mombelli et al., 1992); Not prevalent (Perdikogianni et al., 2009; Machorowska-Pieniążek et al., 2013) |  | |  | | | (Perdikogianni et al., 2009) |
|  |  |  | N/A (Mombelli et al., 1992; Machorowska-Pieniążek et al., 2013) | | | | | |
|  | *Capnocytophaga sputigena* | N/A (Iurovschi et al., 2020) | N/A (Iurovschi et al., 2020) | | | | | |
| 6 | *Eubacterium brachy* | N/A (Iurovschi et al., 2020) | N/A (Iurovschi et al., 2020) | | | | | |
|  | *Eubacterium limosum* | N/A (Iurovschi et al., 2020) | N/A (Iurovschi et al., 2020) | | | | | |
|  | *Eubacterium nodatum* | N/A (Iurovschi et al., 2020) | N/A (Iurovschi et al., 2020) | | | | | |
|  | *Eubacterium saburreum* | N/A (Iurovschi et al., 2020) | N/A (Iurovschi et al., 2020) | | | | | |
|  | *Eubacterium* spp. | Not prevalent (Perdikogianni et al., 2009; Machorowska-Pieniążek et al., 2013) |  | |  | | | (Perdikogianni et al., 2009) |
|  |  |  | N/A (Machorowska-Pieniążek et al., 2013) | | | | | |
| 6 | *Neisseria gonorrhoeae* | N/A (Iurovschi et al., 2020) | N/A (Iurovschi et al., 2020) | | | | | |
|  | *Neisseria meningitidis* | N/A (Iurovschi et al., 2020) | N/A (Iurovschi et al., 2020) | | | | | |
|  | *Neisseria mucosa* | N/A (Iurovschi et al., 2020) | N/A (Iurovschi et al., 2020) | | | | | |
|  | *Neisseria polysaccharea* | N/A (Iurovschi et al., 2020) | N/A (Iurovschi et al., 2020) | | | | | |
|  | *Neisseria* spp. | Highly prevalent (Machorowska-Pieniążek et al., 2013); Not prevalent (Perdikogianni et al., 2009) |  | | (Perdikogianni et al., 2009) | | |  |
|  |  |  | N/A (Machorowska-Pieniążek et al., 2013) | | | | | |
| 6 | *Porphyromonas asaccharolytica* | Not prevalent (Perdikogianni et al., 2009) |  | | (Perdikogianni et al., 2009) | | |  |
|  | *Porphyromonas endodontalis* | N/A (Iurovschi et al., 2020) | N/A (Iurovschi et al., 2020) | | | | | |
|  | *Porphyromonas gingivalis* | Not prevalent (Costa et al., 2003; Perdikogianni et al., 2009); N/A (Iurovschi et al., 2020; Passinato Gheller et al., 2021) |  | | (Passinato Gheller et al., 2021) | | | (Perdikogianni et al., 2009) |
|  |  |  | N/A (Costa et al., 2003; Iurovschi et al., 2020) | | | | | |
| 6 | *Veillonella dispar* | N/A (Iurovschi et al., 2020) | N/A (Iurovschi et al., 2020) | | | | | |
|  | *Veillonella parvula* | Not prevalent (Funahashi et al., 2019); N/A (Iurovschi et al., 2020) |  | |  | | | (Funahashi et al., 2019) |
|  |  |  | N/A (Iurovschi et al., 2020) | | | | | |
|  | *Veillonella* spp. | Moderately prevalent (Mombelli et al., 1992); Somewhat prevalent (Machorowska-Pieniążek et al., 2013); Not prevalent (Perdikogianni et al., 2009) |  | |  | | | (Perdikogianni et al., 2009) |
|  |  |  | N/A (Mombelli et al., 1992; Machorowska-Pieniążek et al., 2013) | | | | | |
| 5 | *Campylobacter gracilis* | N/A (Iurovschi et al., 2020) | N/A (Iurovschi et al., 2020) | | | | | |
|  | *Campylobacter rectus* | Moderately prevalent (Quirynen et al., 2003); N/A (Iurovschi et al., 2020) | N/A (Quirynen et al., 2003; Iurovschi et al., 2020) | | | | | |
|  | *Campylobacter showae* | N/A (Iurovschi et al., 2020) | N/A (Iurovschi et al., 2020) | | | | | |
|  | *Campylobacter* spp. | Not prevalent (Perdikogianni et al., 2009) |  | |  | | | (Perdikogianni et al., 2009) |
| 5 | *Selenomonas artemidis* | Not prevalent (Funahashi et al., 2019) |  | | (Funahashi et al., 2019) | | |  |
|  | *Selenomonas noxia* | Not prevalent (Funahashi et al., 2019); N/A (Iurovschi et al., 2020) |  | |  | | | (Funahashi et al., 2019) |
|  |  |  | N/A (Iurovschi et al., 2020) | | | | | |
|  | *Selenomonas* spp. | Not prevalent (Mombelli et al., 1992; Perdikogianni et al., 2009) |  | |  | | | (Perdikogianni et al., 2009) |
|  |  |  | N/A (Mombelli et al., 1992) | | | | | |
| 4 | *Aggregatibacter actinomycetemcomitans* | N/A (Iurovschi et al., 2020; Passinato Gheller et al., 2021) |  | | | (Passinato Gheller et al., 2021) |  | |
|  |  |  | N/A (Iurovschi et al., 2020) | | | | | |
|  | *Aggregatibacter aphrophilus* | Not prevalent (Funahashi et al., 2019); N/A (Iurovschi et al., 2020) |  | | | (Funahashi et al., 2019) |  | |
|  |  |  | N/A (Iurovschi et al., 2020) | | | | | |
| 4 | *Enterobacter aerogenes* | N/A (Iurovschi et al., 2020) | N/A (Iurovschi et al., 2020) | | | | | |
|  | *Enterobacter cloacae* | N/A (Iurovschi et al., 2020) | N/A (Iurovschi et al., 2020) | | | | | |
|  | *Enterobacter gergoviae* | N/A (Iurovschi et al., 2020) | N/A (Iurovschi et al., 2020) | | | | | |
|  | *Enterobacter kobei* | Not prevalent (Machorowska-Pieniążek et al., 2013) | N/A (Machorowska-Pieniążek et al., 2013) | | | | | |
| 4 | *Enterococcus durans* | Not prevalent (Funahashi et al., 2019) |  | |  | | | (Funahashi et al., 2019) |
|  | *Enterococcus faecalis* | N/A (Iurovschi et al., 2020) | N/A (Iurovschi et al., 2020) | | | | | |
|  | *Enterococcus faecium* | N/A (Iurovschi et al., 2020) | N/A (Iurovschi et al., 2020) | | | | | |
|  | *Enterococcus hirae* | N/A (Iurovschi et al., 2020) | N/A (Iurovschi et al., 2020) | | | | | |
| 4 | *Gemella hemolysans* | Not prevalent (Perdikogianni et al., 2009) | (Perdikogianni et al., 2009) | |  | | |  |
|  | *Gemella morbillorum* | Not prevalent (Perdikogianni et al., 2009; Machorowska-Pieniążek et al., 2013); N/A (Iurovschi et al., 2020) | (Perdikogianni et al., 2009) | |  | | |  |
|  |  |  | N/A (Machorowska-Pieniążek et al., 2013) | | | | | |
|  |  |  | N/A (Iurovschi et al., 2020) | | | | | |
| 4 | *Leptotrichia buccalis* | Not prevalent (Perdikogianni et al., 2009); N/A (Iurovschi et al., 2020) |  | | (Perdikogianni et al., 2009) | | |  |
|  |  |  | N/A (Iurovschi et al., 2020) | | | | | |
|  | *Leptotrichia goodfellowii* | Not prevalent (Funahashi et al., 2019) |  | |  | | | (Funahashi et al., 2019) |
|  | *Leptotrichia hofstadii* | Not prevalent (Funahashi et al., 2019) |  | |  | | | (Funahashi et al., 2019) |
| 4 | *Staphylococcus aureus* | N/A (Iurovschi et al., 2020) | N/A (Iurovschi et al., 2020) | | | | | |
|  | *Staphylococcus epidermidis* | Not prevalent (Perdikogianni et al., 2009); N/A (Iurovschi et al., 2020) |  | | (Perdikogianni et al., 2009) | | |  |
|  |  |  | N/A (Iurovschi et al., 2020) | | | | | |
|  | *Staphylococcus warneri* | N/A (Iurovschi et al., 2020) | N/A (Iurovschi et al., 2020) | | | | | |
| 4 | *Treponema denticola* | N/A (Costa et al., 2003; Iurovschi et al., 2020) | N/A (Costa et al., 2003; Iurovschi et al., 2020) | | | | | |
|  | *Treponema socranskii* | Not prevalent (Funahashi et al., 2019); N/A (Iurovschi et al., 2020) |  |  | | | (Funahashi et al., 2019) | |
|  |  |  | N/A (Iurovschi et al., 2020) | | | | | |
| 3 | *Bacteroides fragilis* | N/A (Iurovschi et al., 2020) | N/A (Iurovschi et al., 2020) | | | | | |
|  | *Bacteroides* spp. | Not prevalent (Perdikogianni et al., 2009; Machorowska-Pieniążek et al., 2013) |  | |  | | | (Perdikogianni et al., 2009) |
|  |  |  | N/A (Machorowska-Pieniążek et al., 2013) | | | | | |
| 3 | *Bifidobacterium dentium* | Not prevalent (Funahashi et al., 2019) |  | | (Funahashi et al., 2019) | | |  |
|  | *Bifidobacterium* spp. | Somewhat prevalent (Machorowska-Pieniążek et al., 2013); Not prevalent (Perdikogianni et al., 2009) |  | |  | | | (Perdikogianni et al., 2009) |
|  |  |  | N/A (Machorowska-Pieniążek et al., 2013) | | | | | |
| 3 | *Eikenella corrodens* | Not prevalent (Mombelli et al., 1992; Perdikogianni et al., 2009); N/A (Iurovschi et al., 2020) |  | | |  | | (Perdikogianni et al., 2009) |
|  |  |  | N/A (Mombelli et al., 1992; Iurovschi et al., 2020) | | | | | |
| 3 | *Haemophilus haemolyticus* | Not prevalent (Funahashi et al., 2019) |  | |  | | | (Funahashi et al., 2019) |
|  | *Haemophilus influenzae* | N/A (Iurovschi et al., 2020) | N/A (Iurovschi et al., 2020) | | | | | |
|  | *Haemophilus* spp. | Not prevalent (Perdikogianni et al., 2009) |  | | (Perdikogianni et al., 2009) | | |  |
| 3 | *Mitsuokella multacida* | Not prevalent (Machorowska-Pieniążek et al., 2013; Funahashi et al., 2019) | (Funahashi et al., 2019) | |  | | |  |
|  |  |  | N/A (Machorowska-Pieniążek et al., 2013) | | | | | |
|  | *Mitsuokella* spp. | N/A (Iurovschi et al., 2020) | N/A (Iurovschi et al., 2020) | |  | | |  |
| 2 | *Parvimonas micra* | Not prevalent (Perdikogianni et al., 2009); N/A (Iurovschi et al., 2020) |  | |  | | | (Perdikogianni et al., 2009) |
|  |  |  | N/A (Iurovschi et al., 2020) | | | | | |
| 2 | *Clostridium* spp. | Not prevalent (Perdikogianni et al., 2009; Machorowska-Pieniążek et al., 2013) |  | | (Perdikogianni et al., 2009) | | |  |
|  |  |  | N/A (Machorowska-Pieniążek et al., 2013) | | | | | |
| 2 | *Corynebacterium matruchotii* | Not prevalent (Perdikogianni et al., 2009); N/A (Iurovschi et al., 2020) |  | | (Perdikogianni et al., 2009) | | |  |
|  |  |  | N/A (Iurovschi et al., 2020) | | | | | |
| 2 | *Klebsiella oxytoca* | Not prevalent (Machorowska-Pieniążek et al., 2013) | N/A (Machorowska-Pieniążek et al., 2013) | | | | | |
|  | *Klebsiella pneumoniae* | Not prevalent (Machorowska-Pieniążek et al., 2013) | N/A (Machorowska-Pieniążek et al., 2013) | | | | | |
| 2 | *Propionibacterium acnes* | N/A (Iurovschi et al., 2020) | N/A (Iurovschi et al., 2020) | | | | | |
|  | *Propionibacterium* spp. | Not prevalent (Perdikogianni et al., 2009) |  | | (Perdikogianni et al., 2009) | | |  |
| 2 | *Rothia dentocariosa* | Not prevalent (Perdikogianni et al., 2009); N/A (Iurovschi et al., 2020) |  | | (Perdikogianni et al., 2009) | | |  |
|  |  |  | N/A (Iurovschi et al., 2020) | | | | | |
| 2 | *Tannerella forsythia* | N/A (Iurovschi et al., 2020; Passinato Gheller et al., 2021) | (Passinato Gheller et al., 2021) (supragingival) | | (Passinato Gheller et al., 2021) (subgingival) | | |  |
|  |  |  | N/A (Iurovschi et al., 2020) | | | | | |
| 2 | *Wolinella* spp. | Not prevalent (Mombelli et al., 1992; Perdikogianni et al., 2009) |  | | (Perdikogianni et al., 2009) | | |  |
|  |  |  | N/A (Mombelli et al., 1992) | | | | | |
| 1 | *Anaeroglobus geminatus* | Not prevalent (Funahashi et al., 2019) |  | | (Funahashi et al., 2019) | | |  |
| 1 | *Atopobium rimae* | N/A (Iurovschi et al., 2020) | N/A (Iurovschi et al., 2020) | | | | | |
| 1 | *Bilophila wadsworthia* | Not prevalent (Perdikogianni et al., 2009) |  | | (Perdikogianni et al., 2009) | | |  |
| 1 | *Catonella morbi* | Not prevalent (Funahashi et al., 2019) |  | | (Funahashi et al., 2019) | | |  |
| 1 | *Centipeda periodontii* | Not prevalent (Funahashi et al., 2019) |  | |  | | | (Funahashi et al., 2019) |
| 1 | *Dialister invisus* | Not prevalent (Funahashi et al., 2019) |  | |  | | | (Funahashi et al., 2019) |
| 1 | *Erysipelothrix tonsillarum* | Not prevalent (Funahashi et al., 2019) | (Funahashi et al., 2019) | |  | | |  |
| 1 | *Lachnoanaerobaculum saburream* | Not prevalent (Funahashi et al., 2019) |  | |  | | | (Funahashi et al., 2019) |
| 1 | *Mobiluncus curtisii* | N/A (Iurovschi et al., 2020) | N/A (Iurovschi et al., 2020) | | | | | |
| 1 | *Mycoplasma salivarium* | Not prevalent (Funahashi et al., 2019) |  | | (Funahashi et al., 2019) | | |  |
| 1 | *Nectria haemotococca* | N/A (Iurovschi et al., 2020) | N/A (Iurovschi et al., 2020) | | | | | |
| 1 | *Oribacterium sinus* | Not prevalent (Funahashi et al., 2019) |  | |  | | | (Funahashi et al., 2019) |
| 1 | *Ottowia* spp. | Not prevalent (Funahashi et al., 2019) |  | | (Funahashi et al., 2019) | | |  |
| 1 | *Peptostreptococcus micra* | Somewhat prevalent (Quirynen et al., 2003) | N/A (Quirynen et al., 2003) | | | | | |
| 1 | *Scardovia wiggsiae* | Not prevalent (Funahashi et al., 2019) |  | | (Funahashi et al., 2019) | | |  |
| 1 | *Serratia marcescens* | N/A (Iurovschi et al., 2020) | N/A (Iurovschi et al., 2020) | | | | | |
| 1 | *Shuttleworthia satelles* | Not prevalent (Funahashi et al., 2019) |  | | (Funahashi et al., 2019) | | |  |
| 1 | *Sneathia amnii* | Not prevalent (Funahashi et al., 2019) |  | | (Funahashi et al., 2019) | | |  |
| 1 | *Solobacterium moorei* | Not prevalent (Funahashi et al., 2019) |  | |  | | | (Funahashi et al., 2019) |
| 1 | *Spiroplasma ixodetis* | N/A (Iurovschi et al., 2020) | N/A (Iurovschi et al., 2020) | | | | | |
| 1 | *Stomatobacculum longum* | Not prevalent (Funahashi et al., 2019) | (Funahashi et al., 2019) | |  | | |  |
| 1 | *Stomatococcus* spp. | Not prevalent (Perdikogianni et al., 2009) |  | | (Perdikogianni et al., 2009) | | |  |
| 1 | *Vibrio nereis* | N/A (Iurovschi et al., 2020) | N/A (Iurovschi et al., 2020) | | | | | |

**Supplementary Table S3. Bacterial species identified on the oral mucosa of CL/P and non-CL/P patients.**

| **Genus Frequency** | **Species Name** | **Location** | **Prevalence in CL/P** | **Significant Difference** | | |
| --- | --- | --- | --- | --- | --- | --- |
|  |  |  |  | **Prone to non-CL/P** | **Prone to CL/P** | **Not Prone to Either** |
| 28 | *Streptococcus acidominimus* | Cleft margin and dorsum of the tongue (Machorowska-Pieniążek et al., 2017) | Not prevalent (Machorowska-Pieniążek et al., 2017) | N/A (Machorowska-Pieniążek et al., 2017) | | |
|  | *Streptococcus agalactiae* | Cleft margin and dorsum of the tongue (Machorowska-Pieniążek et al., 2017) | Not prevalent (Machorowska-Pieniążek et al., 2017) | N/A (Machorowska-Pieniążek et al., 2017) | | |
|  | *Streptococcus anginosus* | Alveolar slit (Iurovschi et al., 2020); Cleft margin and dorsum of the tongue (Machorowska-Pieniążek et al., 2017) | Not prevalent (Machorowska-Pieniążek et al., 2017); N/A (Iurovschi et al., 2020) | N/A (Machorowska-Pieniążek et al., 2017; Iurovschi et al., 2020) | | |
|  | *Streptococcus bovis* biovar I | Cleft margin and dorsum of the tongue (Machorowska-Pieniążek et al., 2017) | Somewhat prevalent (Machorowska-Pieniążek et al., 2017) | N/A (Machorowska-Pieniążek et al., 2017) | | |
|  | *Streptococcus constellatus* | Alveolar slit (Iurovschi et al., 2020); Cleft margin and dorsum of the tongue (Machorowska-Pieniążek et al., 2017) | Not prevalent (Machorowska-Pieniążek et al., 2017); N/A (Iurovschi et al., 2020) | N/A (Machorowska-Pieniążek et al., 2017; Iurovschi et al., 2020) | | |
|  | *Streptococcus dysgalactiae* | Cleft margin and dorsum of the tongue (Machorowska-Pieniążek et al., 2017) | Not prevalent (Machorowska-Pieniążek et al., 2017) | N/A (Machorowska-Pieniążek et al., 2017) | | |
|  | *Streptococcus gordonii* | Alveolar slit (Iurovschi et al., 2020) | N/A (Iurovschi et al., 2020) | N/A (Iurovschi et al., 2020) | | |
|  | *Streptococcus intermedius* | Alveolar slit (Iurovschi et al., 2020); Cleft margin and dorsum of the tongue (Machorowska-Pieniążek et al., 2017) | Not prevalent (Machorowska-Pieniążek et al., 2017), N/A (Iurovschi et al., 2020) | N/A (Machorowska-Pieniążek et al., 2017; Iurovschi et al., 2020) | | |
|  | *Streptococcus liquefaciens* | Sublingual (Cocco et al., 2010) | Not prevalent (Cocco et al., 2010) | N/A (Cocco et al., 2010) | | |
|  | *Streptococcus mitis* | Alveolar slit (Iurovschi et al., 2020); Cleft margin and dorsum of the tongue (Machorowska-Pieniążek et al., 2017) | Highly prevalent (Machorowska-Pieniążek et al., 2017); N/A (Iurovschi et al., 2020) | N/A (Machorowska-Pieniążek et al., 2017; Iurovschi et al., 2020) | | |
|  | *Streptococcus mutans* | Alveolar slit (Iurovschi et al., 2020) | N/A (Iurovschi et al., 2020) | N/A (Iurovschi et al., 2020) | | |
|  | *Streptococcus oralis* | Alveolar slit (Iurovschi et al., 2020); Cleft margin and dorsum of the tongue (Machorowska-Pieniążek et al., 2017) | Not prevalent (Machorowska-Pieniążek et al., 2017); N/A (Iurovschi et al., 2020) | N/A (Machorowska-Pieniążek et al., 2017; Iurovschi et al., 2020) | | |
|  | *Streptococcus pneumoniae* | Cleft margin and dorsum of the tongue (Machorowska-Pieniążek et al., 2017) | Not prevalent (Machorowska-Pieniążek et al., 2017) | N/A (Machorowska-Pieniążek et al., 2017) | | |
|  | *Streptococcus pyogenes* | Alveolar slit (Iurovschi et al., 2020) | N/A (Iurovschi et al., 2020) | N/A (Iurovschi et al., 2020) | | |
|  | *Streptococcus salivarius* | Cleft margin and dorsum of the tongue (Machorowska-Pieniążek et al., 2017) | Somewhat prevalent (Machorowska-Pieniążek et al., 2017) | N/A (Machorowska-Pieniążek et al., 2017) | | |
|  | *Streptococcus sanguinis* | Alveolar slit (Iurovschi et al., 2020); Cleft margin and dorsum of the tongue (Machorowska-Pieniążek et al., 2017) | Somewhat prevalent (Machorowska-Pieniążek et al., 2017); N/A (Iurovschi et al., 2020) | N/A (Machorowska-Pieniążek et al., 2017; Iurovschi et al., 2020) | | |
|  | *Streptococcus* spp. | Palatal cleft (Rodrigues et al., 2021); Alveolar bone mucosa (Zhang et al., 2022) | Somewhat prevalent (Rodrigues et al., 2021; Zhang et al., 2022) | N/A (Rodrigues et al., 2021; Zhang et al., 2022) | | |
|  | beta-*Streptococcus*, Group A | Sublingual (Cocco et al., 2010) | Not prevalent (Cocco et al., 2010) | N/A (Cocco et al., 2010) | | |
|  | *Streptococcus uberis* | Cleft margin and dorsum of the tongue (Machorowska-Pieniążek et al., 2017) | Not prevalent (Machorowska-Pieniążek et al., 2017) | N/A (Machorowska-Pieniążek et al., 2017) | | |
|  | *Streptococcus vestibularis* | Cleft margin and dorsum of the tongue (Machorowska-Pieniążek et al., 2017) | Not prevalent (Machorowska-Pieniążek et al., 2017) | N/A (Machorowska-Pieniążek et al., 2017) | | |
|  | *Streptococcus viridans* | Palatal cleft and throat (Ramdial and Madaree, 2019) | Somewhat prevalent (Ramdial and Madaree, 2019) | N/A (Ramdial and Madaree, 2019) | | |
| 15 | *Staphylococcus aureus* | Alveolar slit (Iurovschi et al., 2020) | N/A (Iurovschi et al., 2020) | N/A (Iurovschi et al., 2020) | | |
|  | Methicillin-resistant *Staphylococcus aureus* | Cleft margin and dorsum of the tongue (Machorowska-Pieniążek et al., 2017); Sublingual (Cocco et al., 2010) | Not prevalent (Cocco et al., 2010; Machorowska-Pieniążek et al., 2017) | N/A (Cocco et al., 2010; Machorowska-Pieniążek et al., 2017) | | |
|  | Methicillin-susceptible *Staphylococcus aureus* | Cleft margin and dorsum of the tongue (Machorowska-Pieniążek et al., 2017); Sublingual (Cocco et al., 2010) | Moderately prevalent (Machorowska-Pieniążek et al., 2017); Somewhat prevalent (Cocco et al., 2010) | N/A (Cocco et al., 2010; Machorowska-Pieniążek et al., 2017) | | |
|  | *Staphylococcus epidermidis* | Alveolar slit (Iurovschi et al., 2020); Cleft margin and dorsum of the tongue (Machorowska-Pieniążek et al., 2017); Sublingual (Cocco et al., 2010) | Somewhat prevalent (Machorowska-Pieniążek et al., 2017); Not prevalent (Cocco et al., 2010); N/A (Iurovschi et al., 2020) | N/A (Cocco et al., 2010; Machorowska-Pieniążek et al., 2017; Iurovschi et al., 2020) | | |
|  | *Staphylococcus haemolyticus* | Cleft margin and dorsum of the tongue (Machorowska-Pieniążek et al., 2017) | Not prevalent (Machorowska-Pieniążek et al., 2017) | N/A (Machorowska-Pieniążek et al., 2017) | | |
|  | *Staphylococcus hominis* | Cleft margin and dorsum of the tongue (Machorowska-Pieniążek et al., 2017) | Not prevalent (Machorowska-Pieniążek et al., 2017) | N/A (Machorowska-Pieniążek et al., 2017) | | |
|  | *Staphylococcus lugdunensis* | Cleft margin and dorsum of the tongue (Machorowska-Pieniążek et al., 2017); Sublingual (Cocco et al., 2010) | Not prevalent (Cocco et al., 2010; Machorowska-Pieniążek et al., 2017) | N/A (Cocco et al., 2010; Machorowska-Pieniążek et al., 2017) | | |
|  | *Staphylococcus simulans* | Sublingual (Cocco et al., 2010) | Not prevalent (Cocco et al., 2010) | N/A (Cocco et al., 2010) | | |
|  | *Staphylococcus warneri* | Alveolar slit (Iurovschi et al., 2020) | N/A (Iurovschi et al., 2020) | N/A (Iurovschi et al., 2020) | | |
|  | *Staphylococcus xylosus* | Cleft margin and dorsum of the tongue (Machorowska-Pieniążek et al., 2017) | Not prevalent (Machorowska-Pieniążek et al., 2017) | N/A (Machorowska-Pieniążek et al., 2017) | | |
| 8 | *Actinomyces israelii* | Alveolar slit (Iurovschi et al., 2020) | N/A (Iurovschi et al., 2020) | N/A (Iurovschi et al., 2020) | | |
|  | *Actinomyces gerenseriae* | Alveolar slit (Iurovschi et al., 2020) | N/A (Iurovschi et al., 2020) | N/A (Iurovschi et al., 2020) | | |
|  | *Actinomyces meyeri* | Alveolar slit (Iurovschi et al., 2020) | N/A (Iurovschi et al., 2020) | N/A (Iurovschi et al., 2020) | | |
|  | *Actinomyces naeslundii* | Alveolar slit (Iurovschi et al., 2020) | N/A (Iurovschi et al., 2020) | N/A (Iurovschi et al., 2020) | | |
|  | *Actinomyces odontolyticus* | Alveolar slit (Iurovschi et al., 2020) | N/A (Iurovschi et al., 2020) | N/A (Iurovschi et al., 2020) | | |
|  | *Actinomyces oris* | Alveolar slit (Iurovschi et al., 2020) | N/A (Iurovschi et al., 2020) | N/A (Iurovschi et al., 2020) | | |
|  | *Actinomyces* spp. | Alveolar bone mucosa (Zhang et al., 2022) | Not prevalent (Zhang et al., 2022) | N/A (Zhang et al., 2022) | | |
|  | *Actinomyces viscosus* | Palatal cleft (Mombelli et al., 1992) | Moderately prevalent (Mombelli et al., 1992) | N/A (Mombelli et al., 1992) | | |
| 8 | *Enterobacter aerogenes* | Alveolar slit (Iurovschi et al., 2020); Cleft margin and dorsum of the tongue (Machorowska-Pieniążek et al., 2017) | Not prevalent (Machorowska-Pieniążek et al., 2017); N/A (Iurovschi et al., 2020) | N/A (Machorowska-Pieniążek et al., 2017; Iurovschi et al., 2020) | | |
|  | *Enterobacter asburiae* | Cleft margin and dorsum of the tongue (Machorowska-Pieniążek et al., 2017) | Not prevalent (Machorowska-Pieniążek et al., 2017) | N/A (Machorowska-Pieniążek et al., 2017) | | |
|  | *Enterobacter cloacae* | Alveolar slit (Iurovschi et al., 2020); Cleft margin and dorsum of the tongue (Machorowska-Pieniążek et al., 2017); Sublingual (Cocco et al., 2010) | Not prevalent (Cocco et al., 2010; Machorowska-Pieniążek et al., 2017); N/A (Iurovschi et al., 2020) | N/A (Cocco et al., 2010; Machorowska-Pieniążek et al., 2017; Iurovschi et al., 2020) | | |
|  | *Enterobacter gergoviae* | Alveolar slit (Iurovschi et al., 2020) | N/A (Iurovschi et al., 2020) | N/A (Iurovschi et al., 2020) | | |
|  | *Enterobacter kobei* | Cleft margin and dorsum of the tongue (Machorowska-Pieniążek et al., 2017) | Not prevalent (Machorowska-Pieniążek et al., 2017) | N/A (Machorowska-Pieniążek et al., 2017) | | |
| 7 | *Fusobacterium necrophorum* | Alveolar slit (Iurovschi et al., 2020) | N/A (Iurovschi et al., 2020) | N/A (Iurovschi et al., 2020) | | |
|  | *Fusobacterium nucleatum nucleatum* | Alveolar slit (Iurovschi et al., 2020) | N/A (Iurovschi et al., 2020) | N/A (Iurovschi et al., 2020) | | |
|  | *Fusobacterium nucleatum polymorphum* | Alveolar slit (Iurovschi et al., 2020) | N/A (Iurovschi et al., 2020) | N/A (Iurovschi et al., 2020) | | |
|  | *Fusobacterium nucleatum vincentii* | Alveolar slit (Iurovschi et al., 2020) | N/A (Iurovschi et al., 2020) | N/A (Iurovschi et al., 2020) | | |
|  | *Fusobacterium periodonticum* | Alveolar slit (Iurovschi et al., 2020) | N/A (Iurovschi et al., 2020) | N/A (Iurovschi et al., 2020) | | |
|  | *Fusobacterium* spp. | Palatal cleft (Mombelli et al., 1992); Alveolar bone mucosa (Zhang et al., 2022) | Not prevalent (Mombelli et al., 1992; Zhang et al., 2022) | N/A (Mombelli et al., 1992; Zhang et al., 2022) | | |
| 7 | *Neisseria gonorrhoeae* | Alveolar slit (Iurovschi et al., 2020) | N/A (Iurovschi et al., 2020) | N/A (Iurovschi et al., 2020) | | |
|  | *Neisseria meningitidis* | Alveolar slit (Iurovschi et al., 2020) | N/A (Iurovschi et al., 2020) | N/A (Iurovschi et al., 2020) | | |
|  | *Neisseria mucosa* | Alveolar slit (Iurovschi et al., 2020) | N/A (Iurovschi et al., 2020) | N/A (Iurovschi et al., 2020) | | |
|  | *Neisseria polysaccharea* | Alveolar slit (Iurovschi et al., 2020) | N/A (Iurovschi et al., 2020) | N/A (Iurovschi et al., 2020) | | |
|  | *Neisseria* spp. | Cleft margin and dorsum of the tongue (Machorowska-Pieniążek et al., 2017); Palatal cleft (Rodrigues et al., 2021); Alveolar bone mucosa (Zhang et al., 2022) | Not prevalent (Machorowska-Pieniążek et al., 2017; Rodrigues et al., 2021; Zhang et al., 2022) | N/A (Machorowska-Pieniążek et al., 2017; Rodrigues et al., 2021; Zhang et al., 2022) | | |
| 6 | *Prevotella intermedia* | Alveolar slit (Iurovschi et al., 2020); Palatal cleft (Mombelli et al., 1992) | Not prevalent (Mombelli et al., 1992); N/A (Iurovschi et al., 2020) | N/A (Mombelli et al., 1992; Iurovschi et al., 2020) | | |
|  | *Prevotella melaninogenica* | Alveolar slit (Iurovschi et al., 2020) | N/A (Iurovschi et al., 2020) | N/A (Iurovschi et al., 2020) | | |
|  | *Prevotella melaninogenica*/  *loeschi/denticola* | Palatal cleft (Mombelli et al., 1992) | Not prevalent (Mombelli et al., 1992) | N/A (Mombelli et al., 1992) | | |
|  | *Prevotella nigrescens* | Alveolar slit (Iurovschi et al., 2020) | N/A (Iurovschi et al., 2020) | N/A (Iurovschi et al., 2020) | | |
|  | *Prevotella* spp. | Alveolar bone mucosa (Zhang et al., 2022) | Not prevalent (Zhang et al., 2022) | N/A (Zhang et al., 2022) | | |
| 5 | *Capnocytophaga gingivalis* | Alveolar slit (Iurovschi et al., 2020) | N/A (Iurovschi et al., 2020) | N/A (Iurovschi et al., 2020) | | |
|  | *Capnocytophaga ochracea* | Alveolar slit (Iurovschi et al., 2020) | N/A (Iurovschi et al., 2020) | N/A (Iurovschi et al., 2020) | | |
|  | *Capnocytophaga* spp. | Palatal cleft (Mombelli et al., 1992); Alveolar bone mucosa (Zhang et al., 2022) | Not prevalent (Mombelli et al., 1992; Zhang et al., 2022) | N/A (Mombelli et al., 1992; Zhang et al., 2022) | | |
|  | *Capnocytophaga sputigena* | Alveolar slit (Iurovschi et al., 2020) | N/A (Iurovschi et al., 2020) | N/A (Iurovschi et al., 2020) | | |
| 5 | *Enterococcus faecalis* | Alveolar slit (Iurovschi et al., 2020);  Sublingual (Cocco et al., 2010) | Not prevalent (Cocco et al., 2010); N/A (Iurovschi et al., 2020) | N/A (Cocco et al., 2010; Iurovschi et al., 2020) | | |
|  | *Enterococcus faecium* | Alveolar slit (Iurovschi et al., 2020) | N/A (Iurovschi et al., 2020) | N/A (Iurovschi et al., 2020) | | |
|  | *Enterococcus hirae* | Alveolar slit (Iurovschi et al., 2020) | N/A (Iurovschi et al., 2020) | N/A (Iurovschi et al., 2020) | | |
|  | *Enterococcus* spp. | Cleft margin and dorsum of the tongue (Machorowska-Pieniążek et al., 2017) | Not prevalent (Machorowska-Pieniążek et al., 2017) | N/A (Machorowska-Pieniążek et al., 2017) | | |
| 5 | *Gemella hemolysans* | Cleft margin and dorsum of the tongue (Machorowska-Pieniążek et al., 2017) | Not prevalent (Machorowska-Pieniążek et al., 2017) | N/A (Machorowska-Pieniążek et al., 2017) | | |
|  | *Gemella morbillorum* | Alveolar slit (Iurovschi et al., 2020); Cleft margin and dorsum of the tongue (Machorowska-Pieniążek et al., 2017) | Somewhat prevalent (Machorowska-Pieniążek et al., 2017); N/A (Iurovschi et al., 2020) | N/A (Machorowska-Pieniążek et al., 2017; Iurovschi et al., 2020) | | |
|  | *Gemella* spp. | Palatal cleft (Rodrigues et al., 2021); Alveolar bone mucosa (Zhang et al., 2022) | Not prevalent (Rodrigues et al., 2021; Zhang et al., 2022) | N/A (Rodrigues et al., 2021; Zhang et al., 2022) | | |
| 5 | *Porphyromonas endodontalis* | Alveolar slit (Iurovschi et al., 2020) | N/A (Iurovschi et al., 2020) | N/A (Iurovschi et al., 2020) | | |
|  | *Porphyromonas gingivalis* | Alveolar slit (Iurovschi et al., 2020); Palatal cleft (Mombelli et al., 1992) | Not prevalent (Mombelli et al., 1992); N/A (Iurovschi et al., 2020) | N/A (Mombelli et al., 1992; Iurovschi et al., 2020) | | |
|  | *Porphyromonas* spp. | Palatal cleft (Rodrigues et al., 2021); Alveolar bone mucosa (Zhang et al., 2022) | Not prevalent (Rodrigues et al., 2021; Zhang et al., 2022) | N/A (Rodrigues et al., 2021; Zhang et al., 2022) | | |
| 5 | *Veillonella dispar* | Alveolar slit (Iurovschi et al., 2020) | N/A (Iurovschi et al., 2020) | N/A (Iurovschi et al., 2020) | | |
|  | *Veillonella parvula* | Alveolar slit (Iurovschi et al., 2020) | N/A (Iurovschi et al., 2020) | N/A (Iurovschi et al., 2020) | | |
|  | *Veillonella* spp. | Palatal cleft (Mombelli et al., 1992; Rodrigues et al., 2021); Alveolar bone mucosa (Zhang et al., 2022) | Somewhat prevalent (Mombelli et al., 1992); Not prevalent (Rodrigues et al., 2021; Zhang et al., 2022) | N/A (Mombelli et al., 1992; Rodrigues et al., 2021; Zhang et al., 2022) | | |
| 4 | *Aggregatibacter actinomycetemcomitans* (*Actinobacillus actinomycetemcomitans*) | Alveolar slit (Iurovschi et al., 2020); Palatal cleft (Mombelli et al., 1992) | Not prevalent (Mombelli et al., 1992); N/A (Iurovschi et al., 2020) | N/A (Mombelli et al., 1992; Iurovschi et al., 2020) | | |
|  | *Aggregatibacter aphrophilus* | Alveolar slit (Iurovschi et al., 2020) | N/A (Iurovschi et al., 2020) | N/A (Iurovschi et al., 2020) | | |
|  | *Aggregatibacter* spp. | Alveolar bone mucosa (Zhang et al., 2022) | Not prevalent (Zhang et al., 2022) | N/A (Zhang et al., 2022) | | |
| 4 | *Eubacterium brachy* | Alveolar slit (Iurovschi et al., 2020) | N/A (Iurovschi et al., 2020) | N/A (Iurovschi et al., 2020) | | |
|  | *Eubacterium limosum* | Alveolar slit (Iurovschi et al., 2020) | N/A (Iurovschi et al., 2020) | N/A (Iurovschi et al., 2020) | | |
|  | *Eubacterium nodatum* | Alveolar slit (Iurovschi et al., 2020) | N/A (Iurovschi et al., 2020) | N/A (Iurovschi et al., 2020) | | |
|  | *Eubacterium saburreum* | Alveolar slit (Iurovschi et al., 2020) | N/A (Iurovschi et al., 2020) | N/A (Iurovschi et al., 2020) | | |
| 4 | *Klebsiella oxytoca* | Cleft margin and dorsum of the tongue (Machorowska-Pieniążek et al., 2017); Sublingual (Cocco et al., 2010) | Not prevalent (Cocco et al., 2010; Machorowska-Pieniążek et al., 2017) | N/A (Cocco et al., 2010; Machorowska-Pieniążek et al., 2017) | | |
|  | *Klebsiella pneumoniae* | Cleft margin and dorsum of the tongue (Machorowska-Pieniążek et al., 2017); Sublingual (Cocco et al., 2010) | Moderately prevalent (Cocco et al., 2010); Somewhat prevalent (Machorowska-Pieniążek et al., 2017) | N/A (Cocco et al., 2010; Machorowska-Pieniążek et al., 2017) | | |
| 3 | *Campylobacter gracilis* | Alveolar slit (Iurovschi et al., 2020) | N/A (Iurovschi et al., 2020) | N/A (Iurovschi et al., 2020) | | |
|  | *Campylobacter rectus* | Alveolar slit (Iurovschi et al., 2020) | N/A (Iurovschi et al., 2020) | N/A (Iurovschi et al., 2020) | | |
|  | *Campylobacter showae* | Alveolar slit (Iurovschi et al., 2020) | N/A (Iurovschi et al., 2020) | N/A (Iurovschi et al., 2020) | | |
| 3 | *Lactobacillus acidophilus* | Alveolar slit (Iurovschi et al., 2020) | N/A (Iurovschi et al., 2020) | N/A (Iurovschi et al., 2020) | | |
|  | *Lactobacillus fermentum* | Alveolar slit (Iurovschi et al., 2020) | N/A (Iurovschi et al., 2020) | N/A (Iurovschi et al., 2020) | | |
|  | *Lactobacillus* spp. | Cleft margin and dorsum of the tongue (Machorowska-Pieniążek et al., 2017) | Not prevalent (Machorowska-Pieniążek et al., 2017) | N/A (Machorowska-Pieniążek et al., 2017) | | |
| 3 | *Leptotrichia buccalis* | Alveolar slit (Iurovschi et al., 2020) | N/A (Iurovschi et al., 2020) | N/A (Iurovschi et al., 2020) | | |
|  | *Leptotrichia* spp. | Palatal cleft (Rodrigues et al., 2021); Alveolar bone mucosa (Zhang et al., 2022) | Not prevalent (Rodrigues et al., 2021; Zhang et al., 2022) | N/A (Rodrigues et al., 2021; Zhang et al., 2022) | | |
| 3 | *Rothia dentocariosa* | Alveolar slit (Iurovschi et al., 2020) | N/A (Iurovschi et al., 2020) | N/A (Iurovschi et al., 2020) | | |
|  | *Rothia* spp. | Palatal cleft (Rodrigues et al., 2021); Alveolar bone mucosa (Zhang et al., 2022) | Not prevalent (Rodrigues et al., 2021; Zhang et al., 2022) | N/A (Rodrigues et al., 2021; Zhang et al., 2022) | | |
| 3 | *Selenomonas noxia* | Alveolar slit (Iurovschi et al., 2020) | N/A (Iurovschi et al., 2020) | N/A (Iurovschi et al., 2020) | | |
|  | *Selenomonas* spp. | Palatal cleft (Mombelli et al., 1992); Alveolar bone mucosa (Zhang et al., 2022) | Not prevalent (Mombelli et al., 1992; Zhang et al., 2022) | N/A (Mombelli et al., 1992; Zhang et al., 2022) | | |
| 3 | *Serratia fonticola* | Cleft margin and dorsum of the tongue (Machorowska-Pieniążek et al., 2017) | Not prevalent (Machorowska-Pieniążek et al., 2017) | N/A (Machorowska-Pieniążek et al., 2017) | | |
|  | *Serratia liquefaciens* | Cleft margin and dorsum of the tongue (Machorowska-Pieniążek et al., 2017) | Not prevalent (Machorowska-Pieniążek et al., 2017) | N/A (Machorowska-Pieniążek et al., 2017) | | |
|  | *Serratia marcescens* | Alveolar slit (Iurovschi et al., 2020) | N/A (Iurovschi et al., 2020) | N/A (Iurovschi et al., 2020) | | |
| 3 | *Treponema denticola* | Alveolar slit (Iurovschi et al., 2020) | N/A (Iurovschi et al., 2020) | N/A (Iurovschi et al., 2020) | | |
|  | *Treponema socranskii* | Alveolar slit (Iurovschi et al., 2020) | N/A (Iurovschi et al., 2020) | N/A (Iurovschi et al., 2020) | | |
|  | *Treponema* spp. | Alveolar bone mucosa (Zhang et al., 2022) | Not prevalent (Zhang et al., 2022) | N/A (Zhang et al., 2022) | | |
| 2 | *Acinetobacter baumannii* | Cleft margin and dorsum of the tongue (Machorowska-Pieniążek et al., 2017) | Not prevalent (Machorowska-Pieniążek et al., 2017) | N/A (Machorowska-Pieniążek et al., 2017) | | |
|  | *Acinetobacter lwoffii* | Cleft margin and dorsum of the tongue (Machorowska-Pieniążek et al., 2017) | Not prevalent (Machorowska-Pieniążek et al., 2017) | N/A (Machorowska-Pieniążek et al., 2017) | | |
| 2 | *Atopobium rimae* | Alveolar slit (Iurovschi et al., 2020) | N/A (Iurovschi et al., 2020) | N/A (Iurovschi et al., 2020) | | |
|  | *Atopobium* | Alveolar bone mucosa (Zhang et al., 2022) | Not prevalent (Zhang et al., 2022) | N/A (Zhang et al., 2022) | | |
| 2 | *Eikenella corrodens* | Alveolar slit (Iurovschi et al., 2020); Palatal cleft (Mombelli et al., 1992) | Not prevalent (Mombelli et al., 1992); N/A (Iurovschi et al., 2020) | N/A (Mombelli et al., 1992; Iurovschi et al., 2020) | | |
| 2 | *Escherichia coli* | Cleft margin and dorsum of the tongue (Machorowska-Pieniążek et al., 2017); Sublingual (Cocco et al., 2010) | Not prevalent (Cocco et al., 2010; Machorowska-Pieniążek et al., 2017) | N/A (Cocco et al., 2010; Machorowska-Pieniążek et al., 2017) | | |
| 2 | *Granulicatella* spp. | Palatal cleft (Rodrigues et al., 2021); Alveolar bone mucosa (Zhang et al., 2022) | Not prevalent (Rodrigues et al., 2021; Zhang et al., 2022) | N/A (Rodrigues et al., 2021; Zhang et al., 2022) | | |
| 2 | *Haemophilus influenzae* | Alveolar slit (Iurovschi et al., 2020) | N/A (Iurovschi et al., 2020) | N/A (Iurovschi et al., 2020) | | |
|  | *Haemophilus* spp. | Alveolar bone mucosa (Zhang et al., 2022) | Not prevalent (Zhang et al., 2022) | N/A (Zhang et al., 2022) | | |
| 2 | *Moraxella* spp. | Cleft margin and dorsum of the tongue (Machorowska-Pieniążek et al., 2017); Alveolar bone mucosa (Zhang et al., 2022) | Not prevalent (Machorowska-Pieniążek et al., 2017; Zhang et al., 2022) | N/A (Machorowska-Pieniążek et al., 2017; Zhang et al., 2022) | | |
| 2 | *Pseudomonas aeruginosa* | Sublingual (Cocco et al., 2010) | Not prevalent (Cocco et al., 2010) | N/A (Cocco et al., 2010) | | |
|  | *Pseudomonas* spp. | Alveolar bone mucosa (Zhang et al., 2022) | Not prevalent (Zhang et al., 2022) | N/A (Zhang et al., 2022) | | |
| 1 | *Abiotrophia* spp. | Alveolar bone mucosa (Zhang et al., 2022) | Not prevalent (Zhang et al., 2022) | N/A (Zhang et al., 2022) | | |
| 1 | *Alloprevotella* spp. | Alveolar bone mucosa (Zhang et al., 2022) | Not prevalent (Zhang et al., 2022) | N/A (Zhang et al., 2022) | | |
| 1 | *Bacteroides fragilis* | Alveolar slit (Iurovschi et al., 2020) | N/A (Iurovschi et al., 2020) | N/A (Iurovschi et al., 2020) | | |
| 1 | *Bifidobacterium* spp. | Alveolar bone mucosa (Zhang et al., 2022) | Not prevalent (Zhang et al., 2022) | N/A (Zhang et al., 2022) | | |
| 1 | *Citrobacter* spp. | Cleft margin and dorsum of the tongue (Machorowska-Pieniążek et al., 2017) | Not prevalent (Machorowska-Pieniążek et al., 2017) | N/A (Machorowska-Pieniążek et al., 2017) | | |
| 1 | *Corynobacterium matruchotii* | Alveolar slit (Iurovschi et al., 2020) | N/A (Iurovschi et al., 2020) | N/A (Iurovschi et al., 2020) | | |
| 1 | *Dialister* spp. | Alveolar bone mucosa (Zhang et al., 2022) | Not prevalent (Zhang et al., 2022) | N/A (Zhang et al., 2022) | | |
| 1 | *Filifactor* spp. | Alveolar bone mucosa (Zhang et al., 2022) | Not prevalent (Zhang et al., 2022) | N/A (Zhang et al., 2022) | | |
| 1 | *Lactococcus lactis* | Cleft margin and dorsum of the tongue (Machorowska-Pieniążek et al., 2017) | Not prevalent (Machorowska-Pieniążek et al., 2017) | N/A (Machorowska-Pieniążek et al., 2017) | | |
| 1 | *Lautropia* spp. | Alveolar bone mucosa (Zhang et al., 2022) | Not prevalent (Zhang et al., 2022) | N/A (Zhang et al., 2022) | | |
| 1 | *Mobiluncus curtissii* | Alveolar slit (Iurovschi et al., 2020) | N/A (Iurovschi et al., 2020) | N/A (Iurovschi et al., 2020) | | |
| 1 | *Nectria haematococca* | Alveolar slit (Iurovschi et al., 2020) | N/A (Iurovschi et al., 2020) | N/A (Iurovschi et al., 2020) | | |
| 1 | *Parvimonas micra* | Alveolar slit (Iurovschi et al., 2020) | N/A (Iurovschi et al., 2020) | N/A (Iurovschi et al., 2020) | | |
| 1 | *Propionibacterium acnes* | Alveolar slit (Iurovschi et al., 2020) | N/A (Iurovschi et al., 2020) | N/A (Iurovschi et al., 2020) | | |
| 1 | *Shuttleworthia* spp. | Alveolar bone mucosa (Zhang et al., 2022) | Not prevalent (Zhang et al., 2022) | N/A (Zhang et al., 2022) | | |
| 1 | *Solobacterium* spp. | Alveolar bone mucosa (Zhang et al., 2022) | Not prevalent (Zhang et al., 2022) | N/A (Zhang et al., 2022) | | |
| 1 | *Spiroplasma ixodetis* | Alveolar slit (Iurovschi et al., 2020) | N/A (Iurovschi et al., 2020) | N/A (Iurovschi et al., 2020) | | |
| 1 | *Tannerella forsythia* | Alveolar slit (Iurovschi et al., 2020) | N/A (Iurovschi et al., 2020) | N/A (Iurovschi et al., 2020) | | |
| 1 | *Vibrio nereis* | Alveolar slit (Iurovschi et al., 2020) | N/A (Iurovschi et al., 2020) | N/A (Iurovschi et al., 2020) | | |
| 1 | *Wollinella* spp. | Palatal cleft (Mombelli et al., 1992) | Not prevalent (Mombelli et al., 1992) | N/A (Mombelli et al., 1992) | | |

**Supplementary Table S4. Bacterial species found on the non-oral mucosa of CL/P and non-CL/P patients.**

| **Genus Frequency** | **Species Name** | **Location** | **Prevalence in CL/P** | **Significant Difference** | | |
| --- | --- | --- | --- | --- | --- | --- |
|  |  |  |  | **Prone to non-CL/P** | **Prone to CL/P** | **Not Prone to Either** |
| 18 | *Staphylococcus aureus* | Nose and/or oropharynx (Thomas et al., 2012); Throat (Rennie et al., 2009); Nose, throat and ear (Chuo and Timmons, 2005); Nose and/or throat (Narinesingh et al., 2011); Nose (Tuna et al., 2008) | Moderately prevalent (Tuna et al., 2008); Somewhat prevalent (Chuo and Timmons, 2005; Narinesingh et al., 2011; Thomas et al., 2012); Not prevalent (Rennie et al., 2009) | N/A (Chuo and Timmons, 2005; Tuna et al., 2008; Rennie et al., 2009; Narinesingh et al., 2011; Thomas et al., 2012) | | |
|  | Methicillin-resistant *Staphylococcus aureus* | Nose (Cocco et al., 2010); Oropharynx (Cocco et al., 2010); Nose, throat and ear (Chuo and Timmons, 2005); Nose, throat and perineum (Bos et al., 2016) | Not prevalent (Chuo and Timmons, 2005; Cocco et al., 2010); N/A (Bos et al., 2016) | N/A (Chuo and Timmons, 2005; Cocco et al., 2010; Bos et al., 2016) | | |
|  | Methicillin-susceptible *Staphylococcus aureus* | Nose (Cocco et al., 2010) | Moderately prevalent (Cocco et al., 2010) | N/A (Cocco et al., 2010) | | |
|  |  | Oropharynx (Cocco et al., 2010) | Somewhat prevalent (Cocco et al., 2010) |  |  |  |
|  | *Staphylococcus epidermidis* | Nose (Cocco et al., 2010) | Somewhat prevalent (Cocco et al., 2010) | N/A (Cocco et al., 2010) | | |
|  |  | Oropharynx (Cocco et al., 2010) | Not prevalent (Cocco et al., 2010) |  |  |  |
|  | *Staphylococcus lugdunensis* | Nose (Cocco et al., 2010); Oropharynx (Cocco et al., 2010) | Not prevalent (Cocco et al., 2010) | N/A (Cocco et al., 2010) | | |
|  | *Staphylococcus simulans* | Nose (Cocco et al., 2010); Oropharynx (Cocco et al., 2010) | Not prevalent (Cocco et al., 2010) | N/A (Cocco et al., 2010) | | |
|  | *Staphylococcus* spp. | Nose (Zhang et al., 2016) | Highly prevalent (Zhang et al., 2016) |  |  | (Zhang et al., 2016) |
| 17 | *Streptococcus liquefaciens* | Nose (Cocco et al., 2010); Oropharynx (Cocco et al., 2010) | Not prevalent (Cocco et al., 2010) | N/A (Cocco et al., 2010) | | |
|  | *Streptococcus millerae* | Nose and/or oropharynx (Thomas et al., 2012) | Not prevalent (Thomas et al., 2012) | N/A (Thomas et al., 2012) | | |
|  | *Streptococcus pneumoniae* | Nose and/or oropharynx (Thomas et al., 2012) | Not prevalent (Thomas et al., 2012) | N/A (Thomas et al., 2012) | | |
|  | *Streptococcus* spp. | Nose (Zhang et al., 2016) | Highly prevalent (Zhang et al., 2016) |  | (Zhang et al., 2016) |  |
|  | *beta*-haemolytic *Streptococcus* spp. | Nose and/or oropharynx (Thomas et al., 2012); Throat (Rennie et al., 2009) | Not prevalent (Rennie et al., 2009; Thomas et al., 2012) | N/A (Rennie et al., 2009; Thomas et al., 2012) | | |
|  | beta-*Streptococcus* spp., Group A | Nose (Cocco et al., 2010); Oropharynx (Cocco et al., 2010); Nose, throat and ear (Chuo and Timmons, 2005); Nose and/or throat (Narinesingh et al., 2011) | Not prevalent (Chuo and Timmons, 2005; Cocco et al., 2010; Narinesingh et al., 2011) | N/A (Chuo and Timmons, 2005; Cocco et al., 2010; Narinesingh et al., 2011) | | |
|  | beta-*Streptococcus* spp., Group B | Nose, throat and ear (Chuo and Timmons, 2005); Nose and/or throat (Narinesingh et al., 2011) | Not prevalent (Chuo and Timmons, 2005; Narinesingh et al., 2011) | N/A (Chuo and Timmons, 2005; Narinesingh et al., 2011) | | |
|  | beta-*Streptococcus* spp., Group C | Nose, throat and ear (Chuo and Timmons, 2005); Nose and/or throat (Narinesingh et al., 2011) | Not prevalent (Chuo and Timmons, 2005; Narinesingh et al., 2011) | N/A (Chuo and Timmons, 2005; Narinesingh et al., 2011) | | |
|  | beta-*Streptococcus* spp., Group G | Nose, throat and ear (Chuo and Timmons, 2005); Nose and/or throat (Narinesingh et al., 2011) | Not prevalent (Chuo and Timmons, 2005; Narinesingh et al., 2011) | N/A (Chuo and Timmons, 2005; Narinesingh et al., 2011) | | |
| 4 | *Klebsiella oxytoca* | Nose (Cocco et al., 2010); Oropharynx (Cocco et al., 2010) | Not prevalent (Cocco et al., 2010) | N/A (Cocco et al., 2010) | | |
|  | *Klebsiella pneumoniae* | Nose (Cocco et al., 2010) | Somewhat prevalent (Cocco et al., 2010) | N/A (Cocco et al., 2010) | | |
|  |  | Oropharynx (Cocco et al., 2010) | Moderately prevalent (Cocco et al., 2010) |  |  |  |
| 3 | *Moraxella catarrhalis* | Nose (Zhang et al., 2016); Nose and/or throat (Narinesingh et al., 2011) | Moderately prevalent (Zhang et al., 2016); Not prevalent (Narinesingh et al., 2011) |  |  | (Narinesingh et al., 2011; Zhang et al., 2016) |
|  | *Moraxella* spp. | Nose and/or oropharynx (Thomas et al., 2012) | Not prevalent (Thomas et al., 2012) | N/A (Thomas et al., 2012) | | |
| 2 | *Enterobacter cloacae* | Nose (Cocco et al., 2010); Oropharynx (Cocco et al., 2010) | Not prevalent (Cocco et al., 2010) | N/A (Cocco et al., 2010) | | |
| 2 | *Enterococcus faecalis* | Nose (Cocco et al., 2010); Oropharynx (Cocco et al., 2010) | Not prevalent (Cocco et al., 2010) | N/A (Cocco et al., 2010) | | |
| 2 | *Escherichia coli* | Nose (Cocco et al., 2010); Oropharynx (Cocco et al., 2010) | Not prevalent (Cocco et al., 2010) | N/A (Cocco et al., 2010) | | |
| 2 | *Haemophilus influenze* | Nose and/or oropharynx (Thomas et al., 2012) | Not prevalent (Thomas et al., 2012) | N/A (Thomas et al., 2012) | | |
|  | *Haemophilus parainfluenzae* | Nose and/or oropharynx (Thomas et al., 2012) | Not prevalent (Thomas et al., 2012) | N/A (Thomas et al., 2012) | | |
| 2 | *Pseudomonas aeruginosa* | Nose (Cocco et al., 2010); Oropharynx (Cocco et al., 2010) | Not prevalent (Cocco et al., 2010) | N/A (Cocco et al., 2010) | | |
| 1 | *Bacillus* spp. | Nose (Zhang et al., 2016) | Not prevalent (Zhang et al., 2016) | (Zhang et al., 2016) |  |  |
| 1 | *Corynebacterium* spp. | Nose (Zhang et al., 2016) | Moderately prevalent (Zhang et al., 2016) |  |  | (Zhang et al., 2016) |
| 1 | *Dolosigranulum* spp. | Nose (Zhang et al., 2016) | Moderately prevalent (Zhang et al., 2016) | (Zhang et al., 2016) |  |  |
| 1 | *Gemella* spp. | Nose (Zhang et al., 2016) | Somewhat prevalent (Zhang et al., 2016) |  |  | (Zhang et al., 2016) |
| 1 | *Lautropia* spp. | Nose (Zhang et al., 2016) | Not prevalent (Zhang et al., 2016) |  |  | (Zhang et al., 2016) |
| 1 | *Neisseria* spp. | Nose (Zhang et al., 2016) | Somewhat prevalent (Zhang et al., 2016) |  |  | (Zhang et al., 2016) |
| 1 | *Rothia* spp. | Nose (Zhang et al., 2016) | Not prevalent (Zhang et al., 2016) |  |  | (Zhang et al., 2016) |

**Supplementary Table S5. Bacterial species found on both the oral and non-oral mucosa of CL/P and non-CL/P patients.**

| **Genus Frequency** | **Species Name** | **Location** | **Prevalence in CL/P** | **Significant Difference** | | |
| --- | --- | --- | --- | --- | --- | --- |
|  |  |  |  | **Prone to non-CL/P** | **Prone to CL/P** | **Not Prone to Either** |
| 20 | *Streptococcus anginosus* | Soft palate and oro-nasopharynx (Roode et al., 2017) | Not prevalent (Roode et al., 2017) | N/A (Roode et al., 2017) | | |
|  | *Streptococcus mitis/oralis* | Soft palate and nasopharynx (Roode et al., 2022); Palatal cleft and nasopharynx (Roode and Bütow, 2018) | Somewhat prevalent (Roode and Bütow, 2018; Roode et al., 2022) | N/A (Roode and Bütow, 2018; Roode et al., 2022) | | |
|  | *Streptococcus parasanguinis* | Soft palate and nasopharynx (Roode et al., 2022); Palatal cleft and nasopharynx (Roode and Bütow, 2018) | Not prevalent (Roode and Bütow, 2018; Roode et al., 2022) | N/A (Roode and Bütow, 2018; Roode et al., 2022) | | |
|  | *Streptococcus pneumoniae* | Palatal cleft and throat (Ramdial and Madaree, 2019); Soft palate and oro-nasopharynx (Roode et al., 2017); Soft palate and nasopharynx (Roode et al., 2022); Palatal cleft and nasopharynx (Roode and Bütow, 2018); Soft palate and nasopharynx (Mÿburgh and Bütow, 2009) | Not prevalent (Mÿburgh and Bütow, 2009; Roode et al., 2017; Roode and Bütow, 2018; Ramdial and Madaree, 2019; Roode et al., 2022) | N/A (Mÿburgh and Bütow, 2009; Roode et al., 2017; Roode and Bütow, 2018; Ramdial and Madaree, 2019; Roode et al., 2022) | | |
|  | *Streptococcus pseudopneumoniae* | Soft palate and nasopharynx (Roode et al., 2022) | Not prevalent (Roode et al., 2022) | N/A (Roode et al., 2022) | | |
|  | *Streptococcus pyogenes* | Soft palate and oro-nasopharynx (Roode et al., 2017) | Not prevalent (Roode et al., 2017) | N/A (Roode et al., 2017) | | |
|  | *Streptococcus salivarius* | Soft palate and nasopharynx (Roode et al., 2022) | Not prevalent (Roode et al., 2022) | N/A (Roode et al., 2022) | | |
|  | *Streptococcus* spp., alpha-haemolytic | Soft palate and oro-nasopharynx (Roode et al., 2017) | Not prevalent (Roode et al., 2017) | N/A (Roode et al., 2017) | | |
|  | beta-*Streptococcus*, Group A | Palatal cleft and throat (Ramdial and Madaree, 2019) | Not prevalent (Ramdial and Madaree, 2019) | N/A (Ramdial and Madaree, 2019) | | |
|  | beta-*Streptococcus*, Group B | Palatal cleft and throat (Ramdial and Madaree, 2019) | Not prevalent (Ramdial and Madaree, 2019) | N/A (Ramdial and Madaree, 2019) | | |
|  | beta-*Streptococcus*, Group C | Palatal cleft and throat (Ramdial and Madaree, 2019) | Not prevalent (Ramdial and Madaree, 2019) | N/A (Ramdial and Madaree, 2019) | | |
|  | beta-*Streptococcus*, Group G | Palatal cleft and throat (Ramdial and Madaree, 2019) | Not prevalent (Ramdial and Madaree, 2019) | N/A (Ramdial and Madaree, 2019) | | |
|  | *Streptococcus viridans* | Palatal cleft and throat (Ramdial and Madaree, 2019); Soft palate and oro-nasopharynx (Roode et al., 2017) | Somewhat prevalent (Ramdial and Madaree, 2019); Not prevalent (Roode et al., 2017) | N/A (Roode et al., 2017; Ramdial and Madaree, 2019) | | |
| 12 | *Enterobacter aerogenes* | Soft palate and oro-nasopharynx (Roode et al., 2017); Soft palate and nasopharynx (Roode et al., 2022); Soft palate and nasopharynx (Mÿburgh and Bütow, 2009) | Not prevalent (Mÿburgh and Bütow, 2009; Roode et al., 2017; 2022) | N/A (Mÿburgh and Bütow, 2009; Roode et al., 2017; 2022) | | |
|  | *Enterobacter agglomerans* | Soft palate and oro-nasopharynx (Roode et al., 2017); Soft palate and nasopharynx (Mÿburgh and Bütow, 2009) | Not prevalent (Mÿburgh and Bütow, 2009; Roode et al., 2017) | N/A (Mÿburgh and Bütow, 2009; Roode et al., 2017) | | |
|  | *Enterobacter cloacae* | Soft palate and oro-nasopharynx (Roode et al., 2017); Soft palate and nasopharynx (Roode et al., 2022); Palatal cleft and nasopharynx (Roode and Bütow, 2018); Soft palate and nasopharynx (Mÿburgh and Bütow, 2009) | Not prevalent (Mÿburgh and Bütow, 2009; Roode et al., 2017; Roode and Bütow, 2018; Roode et al., 2022) | N/A (Mÿburgh and Bütow, 2009; Roode et al., 2017; Roode and Bütow, 2018; Roode et al., 2022) | | |
|  | *Enterobacter gergoviae* | Soft palate and oro-nasopharynx (Roode et al., 2017) | Not prevalent (Roode et al., 2017) | N/A (Roode et al., 2017) | | |
|  | *Enterobacter hormaechei* | Soft palate and nasopharynx (Roode et al., 2022) | Not prevalent (Roode et al., 2022) | N/A (Roode et al., 2022) | | |
|  | *Enterobacter kobei* | Soft palate and nasopharynx (Roode et al., 2022) | Not prevalent (Roode et al., 2022) | N/A (Roode et al., 2022) | | |
| 9 | *Klebsiella oxytoca* | Soft palate and oro-nasopharynx (Roode et al., 2017); Soft palate and nasopharynx (Roode et al., 2022); Palatal cleft and nasopharynx (Roode and Bütow, 2018); Soft palate and nasopharynx (Mÿburgh and Bütow, 2009) | Not prevalent (Mÿburgh and Bütow, 2009; Roode et al., 2017; Roode and Bütow, 2018; Roode et al., 2022) | N/A (Mÿburgh and Bütow, 2009; Roode et al., 2017; Roode and Bütow, 2018; Roode et al., 2022) | | |
|  | *Klebsiella pneumoniae* | Palatal cleft and throat (Ramdial and Madaree, 2019); Soft palate and oro-nasopharynx (Roode et al., 2017); Soft palate and nasopharynx (Roode et al., 2022); Palatal cleft and nasopharynx (Roode and Bütow, 2018); Soft palate and nasopharynx (Mÿburgh and Bütow, 2009) | Moderately prevalent (Roode and Bütow, 2018; Roode et al., 2022); Somewhat prevalent (Roode et al., 2017); Not prevalent (Mÿburgh and Bütow, 2009; Ramdial and Madaree, 2019) | N/A (Mÿburgh and Bütow, 2009; Roode et al., 2017; Roode and Bütow, 2018; Ramdial and Madaree, 2019; Roode et al., 2022) | | |
| 7 | *Haemophilus haemolyticus* | Soft palate and nasopharynx (Roode et al., 2022) | Not prevalent (Roode et al., 2022) | N/A (Roode et al., 2022) | | |
|  | *Haemophilus influenzae* | Soft palate and oro-nasopharynx (Roode et al., 2017); Soft palate and nasopharynx (Roode et al., 2022); Palatal cleft and nasopharynx (Roode and Bütow, 2018); Soft palate and nasopharynx (Mÿburgh and Bütow, 2009) | Somewhat prevalent (Roode and Bütow, 2018; Roode et al., 2022); Not prevalent (Mÿburgh and Bütow, 2009; Roode et al., 2017) | N/A (Mÿburgh and Bütow, 2009; Roode et al., 2017; Roode and Bütow, 2018; Roode et al., 2022) | | |
|  | *Haemophilus parahaemolyticus* | Soft palate and nasopharynx (Roode et al., 2022) | Not prevalent (Roode et al., 2022) | N/A (Roode et al., 2022) | | |
|  | *Haemophilus parainfluenzae* | Soft palate and nasopharynx (Roode et al., 2022) | Not prevalent (Roode et al., 2022) | N/A (Roode et al., 2022) | | |
| 7 | *Staphylococcus aureus* | Palatal cleft and throat (Ramdial and Madaree, 2019); Soft palate and oro-nasopharynx (Roode et al., 2017); Soft palate and nasopharynx (Roode et al., 2022); Palatal cleft and nasopharynx (Roode and Bütow, 2018); Soft palate and nasopharynx (Mÿburgh and Bütow, 2009) | Somewhat prevalent (Mÿburgh and Bütow, 2009; Roode et al., 2017; Roode and Bütow, 2018); Not prevalent (Ramdial and Madaree, 2019; Roode et al., 2022) | N/A (Mÿburgh and Bütow, 2009; Roode et al., 2017; Roode and Bütow, 2018; Ramdial and Madaree, 2019; Roode et al., 2022) | | |
|  | *Staphylococcus epidermidis* | Soft palate and oro-nasopharynx (Roode et al., 2017); Soft palate and nasopharynx (Roode et al., 2022) | Not prevalent (Roode et al., 2017; 2022) | N/A (Roode et al., 2017; 2022) | | |
| 6 | *Aeromonas hydrophila* | Soft palate and oro-nasopharynx (Roode et al., 2017); Soft palate and nasopharynx (Mÿburgh and Bütow, 2009) | Not prevalent (Mÿburgh and Bütow, 2009; Roode et al., 2017) | N/A (Mÿburgh and Bütow, 2009; Roode et al., 2017) | | |
|  | *Aeromonas hydrophila/caviae* | Palatal cleft and nasopharynx (Roode and Bütow, 2018) | Not prevalent (Roode and Bütow, 2018) | N/A (Roode and Bütow, 2018) | | |
|  | *Aeromonas hydrophila/caviae/*  *sobria* | Soft palate and nasopharynx (Roode et al., 2022) | Not prevalent (Roode et al., 2022) | N/A (Roode et al., 2022) | | |
|  | *Aeromonas sobria* | Soft palate and oro-nasopharynx (Roode et al., 2017); Soft palate and nasopharynx (Mÿburgh and Bütow, 2009) | Not prevalent (Mÿburgh and Bütow, 2009; Roode et al., 2017) | N/A (Mÿburgh and Bütow, 2009; Roode et al., 2017) | | |
| 5 | *Escherichia coli* | Palatal cleft and throat (Ramdial and Madaree, 2019); Soft palate and oro-nasopharynx (Roode et al., 2017); Soft palate and nasopharynx (Roode et al., 2022); Palatal cleft and nasopharynx (Roode and Bütow, 2018); Soft palate and nasopharynx (Mÿburgh and Bütow, 2009) | Not prevalent (Mÿburgh and Bütow, 2009; Roode et al., 2017; Roode and Bütow, 2018; Ramdial and Madaree, 2019; Roode et al., 2022) | N/A (Mÿburgh and Bütow, 2009; Roode et al., 2017; Roode and Bütow, 2018; Ramdial and Madaree, 2019; Roode et al., 2022) | | |
| 5 | *Pseudomonas aeruginosa* | Soft palate and oro-nasopharynx (Roode et al., 2017); Soft palate and nasopharynx (Roode et al., 2022); Palatal cleft and nasopharynx (Roode and Bütow, 2018) | Not prevalent (Roode et al., 2017; Roode and Bütow, 2018; Roode et al., 2022) | N/A (Roode et al., 2017; Roode and Bütow, 2018; Roode et al., 2022) | | |
|  | *Pseudomonas putida* | Soft palate and nasopharynx (Roode et al., 2022); Palatal cleft and nasopharynx (Roode and Bütow, 2018) | Not prevalent (Roode and Bütow, 2018; Roode et al., 2022) | N/A (Roode and Bütow, 2018; Roode et al., 2022) | | |
| 5 | *Serratia marcescens* | Palatal cleft and throat (Ramdial and Madaree, 2019); Soft palate and oro-nasopharynx (Roode et al., 2017); Soft palate and nasopharynx (Roode et al., 2022); Palatal cleft and nasopharynx (Roode and Bütow, 2018); Soft palate and nasopharynx (Mÿburgh and Bütow, 2009) | Not prevalent (Mÿburgh and Bütow, 2009; Roode et al., 2017; Roode and Bütow, 2018; Ramdial and Madaree, 2019; Roode et al., 2022) | N/A (Mÿburgh and Bütow, 2009; Roode et al., 2017; Roode and Bütow, 2018; Ramdial and Madaree, 2019; Roode et al., 2022) | | |
| 3 | *Acinetobacter baumannii* | Soft palate and oro-nasopharynx (Roode et al., 2017); Soft palate and nasopharynx (Roode et al., 2022); Palatal cleft and nasopharynx (Roode and Bütow, 2018) | Not prevalent (Roode et al., 2017; Roode and Bütow, 2018; Roode et al., 2022) | N/A (Roode et al., 2017; Roode and Bütow, 2018; Roode et al., 2022) | | |
| 3 | *Citrobacter freundii* | Soft palate and nasopharynx (Roode et al., 2022); Palatal cleft and nasopharynx (Roode and Bütow, 2018) | Not prevalent (Roode and Bütow, 2018; Roode et al., 2022) | N/A (Roode and Bütow, 2018; Roode et al., 2022) | | |
|  | *Citrobacter koseri* | Soft palate and nasopharynx (Roode et al., 2022) | Not prevalent (Roode et al., 2022) | N/A (Roode et al., 2022) | | |
| 3 | *Moraxella catarrhalis* | Soft palate and oro-nasopharynx (Roode et al., 2017); Soft palate and nasopharynx (Roode et al., 2022); Soft palate and nasopharynx (Mÿburgh and Bütow, 2009) | Not prevalent (Mÿburgh and Bütow, 2009; Roode et al., 2017; 2022) | N/A (Mÿburgh and Bütow, 2009; Roode et al., 2017; 2022) | | |
| 2 | *Chryseobacterium gleum* | Soft palate and nasopharynx (Roode et al., 2022); Palatal cleft and nasopharynx (Roode and Bütow, 2018) | Not prevalent (Roode and Bütow, 2018; Roode et al., 2022) | N/A (Roode and Bütow, 2018; Roode et al., 2022) | | |
| 2 | *Edwardsiella tarda* | Soft palate and nasopharynx (Roode et al., 2022); Palatal cleft and nasopharynx (Roode and Bütow, 2018) | Not prevalent (Roode and Bütow, 2018; Roode et al., 2022) | N/A (Roode and Bütow, 2018; Roode et al., 2022) | | |
| 2 | *Enterococcus faecalis* | Soft palate and oro-nasopharynx (Roode et al., 2017); Soft palate and nasopharynx (Mÿburgh and Bütow, 2009) | Not prevalent (Roode et al., 2017; Roode and Bütow, 2018) | N/A (Roode et al., 2017; Roode and Bütow, 2018) | | |
| 2 | *Geobacillus thermoglucosidasius* | Soft palate and nasopharynx (Roode et al., 2022); Palatal cleft and nasopharynx (Roode and Bütow, 2018) | Not prevalent (Roode and Bütow, 2018; Roode et al., 2022) | N/A (Roode and Bütow, 2018; Roode et al., 2022) | | |
| 2 | *Neisseria subflava* | Soft palate and nasopharynx (Roode et al., 2022); Palatal cleft and nasopharynx (Roode and Bütow, 2018) | Not prevalent (Roode and Bütow, 2018; Roode et al., 2022) | N/A (Roode and Bütow, 2018; Roode et al., 2022) | | |
| 2 | *Proteus mirabilis* | Soft palate and nasopharynx (Roode et al., 2022); Palatal cleft and nasopharynx (Roode and Bütow, 2018) | Not prevalent (Roode and Bütow, 2018; Roode et al., 2022) | N/A (Roode and Bütow, 2018; Roode et al., 2022) | | |
| 2 | *Saccharomyces cerevisiae* | Soft palate and nasopharynx (Roode et al., 2022); Palatal cleft and nasopharynx (Roode and Bütow, 2018) | Not prevalent (Roode and Bütow, 2018; Roode et al., 2022) | N/A (Roode and Bütow, 2018; Roode et al., 2022) | | |
| 2 | *Stenotrophomonas maltophilia* | Palatal cleft and throat (Ramdial and Madaree, 2019); Soft palate and nasopharynx (Roode et al., 2022) | Not prevalent (Ramdial and Madaree, 2019; Roode et al., 2022) | N/A (Ramdial and Madaree, 2019; Roode et al., 2022) | | |
| 1 | *Kluyvera cryocrescens* | Soft palate and oro-nasopharynx (Roode et al., 2017) | Not prevalent (Roode et al., 2017) | N/A (Roode et al., 2017) | | |

**Supplementary Table S6. Bacterial species in the bodily fluids and secretions of CL/P and non-CL/P patients. **SCOM: suppurative chronic otitis media.**

| **Genus Frequency** | **Species Name** | **Location** | **Prevalence in CL/P** | **Significant Difference** | | |
| --- | --- | --- | --- | --- | --- | --- |
|  |  |  |  | **Prone to non-CL/P** | **Prone to CL/P** | **Not Prone to Either** |
| 35 | *Streptococcus gordonii* | Saliva (Sundell et al., 2018) | Highly prevalent (Sundell et al., 2018) | (Sundell et al., 2018) |  |  |
|  | *Streptococcus acidominimus* | Saliva (Arief et al., 2005) | Not prevalent (Arief et al., 2005) | N/A (Arief et al., 2005) | | |
|  | *Streptococcus agalactiae* | SCOM secretion (Jousimies-Somer et al., 1986) | Not prevalent (Jousimies-Somer et al., 1986) | N/A (Jousimies-Somer et al., 1986) | | |
|  | *Streptococcus bovis* biovar 2 | Saliva (Arief et al., 2005) | Not prevalent (Arief et al., 2005) | N/A (Arief et al., 2005) | | |
|  | *Streptococcus intermedius* | Saliva (Arief et al., 2005) | Not prevalent (Arief et al., 2005) | N/A (Arief et al., 2005) | | |
|  | *Streptococcus mitis* | Saliva (Sundell et al., 2018); Saliva (Arief et al., 2005) | Highly (Sundell et al., 2018); Not prevalent (Arief et al., 2005) | (Sundell et al., 2018) |  |  |
|  |  |  |  | N/A (Arief et al., 2005) | | |
|  | *Streptococcus mitis* biovar 1 | Saliva (Arief et al., 2005) | Somewhat prevalent (Arief et al., 2005) | N/A (Arief et al., 2005) | | |
|  | *Streptococcus mitis* biovar 2 | Saliva (Arief et al., 2005) | Not prevalent (Arief et al., 2005) | N/A (Arief et al., 2005) | | |
|  | *Streptococcus mutans* | Saliva (Bokhout et al., 1996; de Soet et al., 1998; van Loveren et al., 1998; Arief et al., 2005; Cheng et al., 2007; Parapanisiou et al., 2009; Antoszewska et al., 2010; Cildir et al., 2012; Ritthagol et al., 2014; Shashni et al., 2015; Sundell et al., 2015; Sundell et al., 2018; Durhan et al., 2019; Hassani et al., 2020; Chaudhari et al., 2021) | Highly prevalent (de Soet et al., 1998; Cheng et al., 2007; Parapanisiou et al., 2009; Cildir et al., 2012; Shashni et al., 2015; Chaudhari et al., 2021); Moderately prevalent (Bokhout et al., 1996; Antoszewska et al., 2010; Sundell et al., 2018); Not prevalent (van Loveren et al., 1998; Arief et al., 2005; Durhan et al., 2019); N/A (Ritthagol et al., 2014; Sundell et al., 2015; Hassani et al., 2020) |  | (Shashni et al., 2015) | (van Loveren et al., 1998; Cheng et al., 2007; Parapanisiou et al., 2009; Antoszewska et al., 2010; Sundell et al., 2018; Durhan et al., 2019; Chaudhari et al., 2021) |
|  |  |  |  | N/A (Bokhout et al., 1996; de Soet et al., 1998; Arief et al., 2005; Cildir et al., 2012; Ritthagol et al., 2014; Hassani et al., 2020) | | |
|  | *Streptococcus oralis* | Saliva (Arief et al., 2005) | Somewhat prevalent (Arief et al., 2005) | N/A (Arief et al., 2005) | | |
|  | *Streptococcus pneumoniae* | SCOM secretion (Jousimies-Somer et al., 1986) | Somewhat prevalent (Jousimies-Somer et al., 1986) | N/A (Jousimies-Somer et al., 1986) | | |
|  | *Streptococcus pyogenes* | SCOM secretion (Weckwerth et al., 2009) | Not prevalent (Weckwerth et al., 2009) | N/A (Weckwerth et al., 2009) | | |
|  | *Streptococcus salivarius* | Saliva (Sundell et al., 2018); Saliva (Arief et al., 2005) | Somewhat prevalent (Arief et al., 2005; Sundell et al., 2018) | (Sundell et al., 2018) |  |  |
|  |  |  |  | N/A (Arief et al., 2005) | | |
|  | *Streptococcus sanguinis* | Saliva (Arief et al., 2005) | Somewhat prevalent (Arief et al., 2005) | N/A (Arief et al., 2005) | | |
|  | *Streptococcus* spp. | Saliva (Liu et al., 2016; Zhang et al., 2016; Zhang et al., 2022) | Highly prevalent (Zhang et al., 2016); Somewhat prevalent (Zhang et al., 2022); Not prevalent (Liu et al., 2016) |  |  | (Zhang et al., 2016) |
|  |  |  |  | N/A (Liu et al., 2016; Zhang et al., 2022) | | |
|  | *Streptococcus viridans* | SCOM secretion (Jousimies-Somer et al., 1986; Weckwerth et al., 2009) | Not prevalent (Jousimies-Somer et al., 1986; Weckwerth et al., 2009) | N/A (Jousimies-Somer et al., 1986; Weckwerth et al., 2009) | | |
| 15 | *Lactobacillus acidophilus* | Saliva (Antoszewska et al., 2010) | Moderately prevalent (Antoszewska et al., 2010) |  |  | (Antoszewska et al., 2010) |
|  | *Lactobacillus casei* | Saliva (Sundell et al., 2018) | Not prevalent (Sundell et al., 2018) |  |  | (Sundell et al., 2018) |
|  | *Lactobacillus salivarius* | Saliva (Sundell et al., 2018) | Not prevalent (Sundell et al., 2018) |  |  | (Sundell et al., 2018) |
|  | *Lactobacillus* spp. | Saliva (Bokhout et al., 1996; van Loveren et al., 1998; Cheng et al., 2007; Parapanisiou et al., 2009; Cildir et al., 2012; Ritthagol et al., 2014; Shashni et al., 2015; Sundell et al., 2015; Durhan et al., 2019; Hassani et al., 2020; Chaudhari et al., 2021); Feces (Vieira et al., 2013) | Highly prevalent (Parapanisiou et al., 2009; Cildir et al., 2012; Sundell et al., 2015; Chaudhari et al., 2021); Moderately prevalent (Cheng et al., 2007); Somewhat prevalent (Shashni et al., 2015; Durhan et al., 2019); Not prevalent (Bokhout et al., 1996; van Loveren et al., 1998); N/A (Vieira et al., 2013; Ritthagol et al., 2014; Hassani et al., 2020) |  | (Sundell et al., 2015; Durhan et al., 2019) | (van Loveren et al., 1998; Cheng et al., 2007; Parapanisiou et al., 2009; Shashni et al., 2015; Chaudhari et al., 2021) |
|  |  |  |  | N/A (Bokhout et al., 1996; Cildir et al., 2012; Vieira et al., 2013; Ritthagol et al., 2014; Hassani et al., 2020) | | |
| 12 | *Staphylococcus aureus* | Saliva (Arief et al., 2005; Tuna et al., 2008; Durhan et al., 2019); SCOM secretion (Jousimies-Somer et al., 1986; Weckwerth et al., 2009; Weckwerth et al., 2014) | Moderately prevalent (Arief et al., 2005; Tuna et al., 2008); Somewhat prevalent (Weckwerth et al., 2014); Not prevalent (Jousimies-Somer et al., 1986; Weckwerth et al., 2009; Durhan et al., 2019) |  |  | (Durhan et al., 2019) |
|  |  |  |  | N/A (Jousimies-Somer et al., 1986; Arief et al., 2005; Tuna et al., 2008; Weckwerth et al., 2009; Weckwerth et al., 2014) | | |
|  | *Staphylococcus aureus*, coagulase-positive | Blood (Adeyemo et al., 2013) | Not prevalent (Adeyemo et al., 2013) | N/A (Adeyemo et al., 2013) | | |
|  | *Staphylococcus epidermidis* | SCOM secretion (Jousimies-Somer et al., 1986) | Somewhat prevalent (Jousimies-Somer et al., 1986) | N/A (Jousimies-Somer et al., 1986) | | |
|  | *Staphylococcus intermedius* | Saliva (Arief et al., 2005) | Not prevalent (Arief et al., 2005) | N/A (Arief et al., 2005) | | |
|  | *Staphylococcus* spp. | Saliva (Zhang et al., 2016) | Not prevalent (Zhang et al., 2016) |  |  | (Zhang et al., 2016) |
|  | *Staphylococcus* spp., coagulase-negative | Blood (Adeyemo et al., 2013) | Somewhat prevalent (Adeyemo et al., 2013) | N/A (Adeyemo et al., 2013) | | |
|  | *Staphylococcus viridans* | SCOM secretion (Weckwerth et al., 2009) | Not prevalent (Weckwerth et al., 2009) | N/A (Weckwerth et al., 2009) | | |
| 6 | *Prevotella intermedia* | Saliva (Liu et al., 2016); Blood (Adeyemo et al., 2013) | Not prevalent (Adeyemo et al., 2013; Liu et al., 2016) | N/A (Adeyemo et al., 2013; Liu et al., 2016) | | |
|  | *Prevotella melaninogenica* | Saliva (Liu et al., 2016) | Not prevalent (Liu et al., 2016) | N/A (Liu et al., 2016) | | |
|  | *Prevotella nigrescens* | Saliva (Liu et al., 2016) | Not prevalent (Liu et al., 2016) | N/A (Liu et al., 2016) | | |
|  | *Prevotella* spp. | Saliva (Liu et al., 2016; Zhang et al., 2022) | Not prevalent (Liu et al., 2016; Zhang et al., 2022) | N/A (Liu et al., 2016; Zhang et al., 2022) | | |
| 5 | *Gemella hemolysans* | Saliva (Arief et al., 2005) | Not prevalent (Arief et al., 2005) | N/A (Arief et al., 2005) | | |
|  | *Gemella morbillorum* | Saliva (Arief et al., 2005) | Not prevalent (Arief et al., 2005) | N/A (Arief et al., 2005) | | |
|  | *Gemella* spp. | Saliva (Liu et al., 2016; Zhang et al., 2022); Saliva (Zhang et al., 2016) | Highly prevalent (Zhang et al., 2016); Not prevalent (Liu et al., 2016; Zhang et al., 2022) |  |  | (Zhang et al., 2016) |
|  |  |  |  | N/A (Liu et al., 2016; Zhang et al., 2022) | | |
| 4 | *Bacteroides* spp. | Feces (Vieira et al., 2013) | N/A (Vieira et al., 2013) | N/A (Vieira et al., 2013) | | |
|  | *Bacteroides stercoris* | Blood (Adeyemo et al., 2013) | Not prevalent (Adeyemo et al., 2013) | N/A (Adeyemo et al., 2013) | | |
|  | *Bacteroides fragilis* | SCOM secretion (Weckwerth et al., 2009; Weckwerth et al., 2014) | Not prevalent (Weckwerth et al., 2009; Weckwerth et al., 2014) | N/A (Weckwerth et al., 2009; Weckwerth et al., 2014) | | |
| 4 | *Enterobacter aerogenes* | SCOM secretion (Weckwerth et al., 2009) | Not prevalent (Weckwerth et al., 2009) | N/A (Weckwerth et al., 2009) | | |
|  | *Enterobacter agglomerans* | SCOM secretion (Weckwerth et al., 2009); Blood (Adeyemo et al., 2013) | Not prevalent (Weckwerth et al., 2009; Adeyemo et al., 2013) | N/A (Weckwerth et al., 2009; Adeyemo et al., 2013) | | |
|  | *Enterobacter cloacae* | Blood (Adeyemo et al., 2013) | Not prevalent (Adeyemo et al., 2013) | N/A (Adeyemo et al., 2013) | | |
| 4 | *Moraxella catarrhalis* | Saliva (Zhang et al., 2016); SCOM secretion (Jousimies-Somer et al., 1986) | Not prevalent (Zhang et al., 2016); Somewhat prevalent (Jousimies-Somer et al., 1986) |  |  | (Zhang et al., 2016) |
|  |  |  |  | N/A (Jousimies-Somer et al., 1986) | | |
|  | *Moraxella* spp. | Saliva (Liu et al., 2016; Zhang et al., 2022) | Not prevalent (Liu et al., 2016; Zhang et al., 2022) | N/A (Liu et al., 2016; Zhang et al., 2022) | | |
| 4 | *Proteus mirabilis* | SCOM secretion (Weckwerth et al., 2009); Blood (Adeyemo et al., 2013) | Not prevalent (Weckwerth et al., 2009; Adeyemo et al., 2013) | N/A (Weckwerth et al., 2009; Adeyemo et al., 2013) | | |
|  | *Proteus stuartii* | Blood (Adeyemo et al., 2013) | Not prevalent (Adeyemo et al., 2013) | N/A (Adeyemo et al., 2013) | | |
|  | *Proteus vulgaris* | Blood (Adeyemo et al., 2013) | Not prevalent (Adeyemo et al., 2013) | N/A (Adeyemo et al., 2013) | | |
| 4 | *Pseudomonas aeruginosa* | SCOM secretion (Weckwerth et al., 2009; Weckwerth et al., 2014) | Moderately prevalent (Weckwerth et al., 2014); Somewhat prevalent (Weckwerth et al., 2009) | N/A (Weckwerth et al., 2009; Weckwerth et al., 2014) | | |
|  | *Pseudomonas fluorescens* | SCOM secretion (Jousimies-Somer et al., 1986) | Not prevalent (Jousimies-Somer et al., 1986) | N/A (Jousimies-Somer et al., 1986) | | |
|  | *Pseudomonas* spp. | Saliva (Zhang et al., 2022) | Not prevalent (Zhang et al., 2022) | N/A (Zhang et al., 2022) | | |
| 4 | *Veillonella dispar* | Saliva (Liu et al., 2016) | Not prevalent (Liu et al., 2016) | N/A (Liu et al., 2016) | | |
|  | *Veillonella* spp. | Saliva (Zhang et al., 2022) | Not prevalent (Zhang et al., 2022) | N/A (Zhang et al., 2022) | | |
|  | *Veillonella parvula* | Saliva (Liu et al., 2016; Sundell et al., 2018) | Not prevalent (Liu et al., 2016; Sundell et al., 2018) | (Sundell et al., 2018) |  |  |
|  |  |  |  | N/A (Liu et al., 2016) | | |
| 3 | *Actinomyces naeslundi* | Saliva (Sundell et al., 2018) | Moderately prevalent (Sundell et al., 2018) |  |  | (Sundell et al., 2018) |
|  | *Actinomyces odontolyticus* | Saliva (Sundell et al., 2018) | Somewhat prevalent (Sundell et al., 2018) |  |  | (Sundell et al., 2018) |
|  | *Actinomyces* spp. | Saliva (Zhang et al., 2022) | Not prevalent (Zhang et al., 2022) | N/A (Zhang et al., 2022) | | |
| 3 | *Bifidobacterium* spp. | Saliva (Zhang et al., 2022); Feces (Vieira et al., 2013) | Not prevalent (Zhang et al., 2022); N/A (Vieira et al., 2013) | N/A (Vieira et al., 2013; Zhang et al., 2022) | | |
|  | *Bifidobacterium dentium* | Saliva (Sundell et al., 2018) | Not prevalent (Sundell et al., 2018) | (Sundell et al., 2018) |  |  |
| 3 | *Enterococcus faecalis* | SCOM secretion (Weckwerth et al., 2009; Weckwerth et al., 2014) | Not prevalent (Weckwerth et al., 2009; Weckwerth et al., 2014) | N/A (Weckwerth et al., 2009; Weckwerth et al., 2014) | | |
|  | *Enterococcus faecium* | Saliva (Arief et al., 2005) | Not prevalent (Arief et al., 2005) | N/A (Arief et al., 2005) | | |
| 3 | *Fusobacterium nucleatum* | Saliva (Sundell et al., 2018); SCOM secretion (Weckwerth et al., 2014) | Moderately prevalent (Sundell et al., 2018); Not prevalent (Weckwerth et al., 2014) | (Sundell et al., 2018) |  |  |
|  |  |  |  | N/A (Weckwerth et al., 2014) | | |
|  | *Fusobacterium* spp. | Saliva (Zhang et al., 2022) | Not prevalent (Zhang et al., 2022) | N/A (Zhang et al., 2022) | | |
| 3 | *Klebsiella oxytoca* | SCOM secretion (Weckwerth et al., 2009) | Not prevalent (Weckwerth et al., 2009) | N/A (Weckwerth et al., 2009) | | |
|  | *Klebsiella pneumoniae* | SCOM secretion (Weckwerth et al., 2009) | Not prevalent (Weckwerth et al., 2009) | N/A (Weckwerth et al., 2009) | | |
|  | *Klebsiella* spp. | SCOM secretion (Jousimies-Somer et al., 1986) | Not prevalent (Jousimies-Somer et al., 1986) | N/A (Jousimies-Somer et al., 1986) | | |
| 3 | *Lautropia* spp. | Saliva (Liu et al., 2016; Zhang et al., 2016; Zhang et al., 2022) | Moderately prevalent (Zhang et al., 2016); Not prevalent (Liu et al., 2016; Zhang et al., 2022) | (Zhang et al., 2016) |  |  |
|  |  |  |  | N/A (Liu et al., 2016; Zhang et al., 2022) | | |
| 3 | *Neisseria* spp. | Saliva (Liu et al., 2016; Zhang et al., 2016; Zhang et al., 2022) | Highly prevalent (Zhang et al., 2016); Not prevalent (Liu et al., 2016; Zhang et al., 2022) |  |  | (Zhang et al., 2016) |
|  |  |  |  | N/A (Liu et al., 2016; Zhang et al., 2022) | | |
| 3 | *Rothia dentocariosa* | Saliva (Sundell et al., 2018) | Moderately prevalent (Sundell et al., 2018) |  |  | (Sundell et al., 2018) |
|  | *Rothia* spp. | Saliva (Zhang et al., 2016; Zhang et al., 2022) | Moderately prevalent (Zhang et al., 2016); Not prevalent (Zhang et al., 2022) |  |  | (Zhang et al., 2016) |
|  |  |  |  | N/A (Zhang et al., 2022) | | |
|  |  |  |  | N/A (Liu et al., 2016) | | |
| 2 | *Capnocytophaga* spp. | Saliva (Liu et al., 2016; Zhang et al., 2022) | Not prevalent (Liu et al., 2016; Zhang et al., 2022) | N/A (Liu et al., 2016; Zhang et al., 2022) | | |
| 2 | *Citrobacter freundii* | SCOM secretion (Weckwerth et al., 2009); Blood (Adeyemo et al., 2013) | Not prevalent (Weckwerth et al., 2009; Adeyemo et al., 2013) | N/A (Weckwerth et al., 2009; Adeyemo et al., 2013) | | |
| 2 | *Corynebacterium* spp. | Saliva (Zhang et al., 2016); SCOM secretion (Jousimies-Somer et al., 1986) | Not prevalent (Jousimies-Somer et al., 1986; Zhang et al., 2016) |  |  | (Zhang et al., 2016) |
|  |  |  |  | N/A (Jousimies-Somer et al., 1986) | | |
| 2 | *Escherichia coli* | SCOM secretion (Weckwerth et al., 2009); Blood (Adeyemo et al., 2013) | Not prevalent (Weckwerth et al., 2009; Adeyemo et al., 2013) | N/A (Weckwerth et al., 2009; Adeyemo et al., 2013) | | |
| 2 | *Haemophilus* spp. | Saliva (Zhang et al., 2022) | Not prevalent (Zhang et al., 2022) | N/A (Zhang et al., 2022) | | |
|  | *Haemophilus influenzae* | SCOM secretion (Jousimies-Somer et al., 1986) | Not prevalent (Jousimies-Somer et al., 1986) | N/A (Jousimies-Somer et al., 1986) | | |
| 2 | *Porphyromonas* spp. | Saliva (Liu et al., 2016; Zhang et al., 2022) | Not prevalent (Liu et al., 2016; Zhang et al., 2022) | N/A (Liu et al., 2016; Zhang et al., 2022) | | |
| 1 | *Abiotrophia* spp. | Saliva (Zhang et al., 2022) | Not prevalent (Zhang et al., 2022) | N/A (Zhang et al., 2022) | | |
| 1 | *Acetinobacter lwoffii* | Blood (Adeyemo et al., 2013) | Not prevalent (Adeyemo et al., 2013) | N/A (Adeyemo et al., 2013) | | |
| 1 | *Aerococcus viridans* | Saliva (Arief et al., 2005) | Not prevalent (Arief et al., 2005) | N/A (Arief et al., 2005) | | |
| 1 | *Aggregatibacter* spp. | Saliva (Zhang et al., 2022) | Not prevalent (Zhang et al., 2022) | N/A (Zhang et al., 2022) | | |
| 1 | *Alloprevotella* spp. | Saliva (Zhang et al., 2022) | Not prevalent (Zhang et al., 2022) | N/A (Zhang et al., 2022) | | |
| 1 | *Atopobium* spp. | Saliva (Zhang et al., 2022) | Not prevalent (Zhang et al., 2022) | N/A (Zhang et al., 2022) | | |
| 1 | *Bacillus* spp. | Saliva (Zhang et al., 2016) | Not prevalent (Zhang et al., 2016) | (Zhang et al., 2016) |  |  |
| 1 | *Dialister* spp. | Saliva (Zhang et al., 2022) | Not prevalent (Zhang et al., 2022) | N/A (Zhang et al., 2022) | | |
| 1 | *Dolosigranulum* spp. | Saliva (Zhang et al., 2016) | Not prevalent (Zhang et al., 2016) |  |  | (Zhang et al., 2016) |
| 1 | *Filifactor* spp*.* | Saliva (Zhang et al., 2022) | Not prevalent (Zhang et al., 2022) | N/A (Zhang et al., 2022) | | |
| 1 | *Flavobacterium meningosepticum* | Blood (Adeyemo et al., 2013) | Not prevalent (Adeyemo et al., 2013) | N/A (Adeyemo et al., 2013) | | |
| 1 | *Granulicatella* spp. | Saliva (Zhang et al., 2022) | Not prevalent (Zhang et al., 2022) | N/A (Zhang et al., 2022) | | |
| 1 | *Leptotrichia* spp. | Saliva (Zhang et al., 2022) | Not prevalent (Zhang et al., 2022) | N/A (Zhang et al., 2022) | | |
| 1 | *Morganella morganii* | SCOM secretion (Weckwerth et al., 2009) | Not prevalent (Weckwerth et al., 2009) | N/A (Weckwerth et al., 2009) | | |
| 1 | *Peptostreptococcus anaerobius* | SCOM secretion (Weckwerth et al., 2014) | Not prevalent (Weckwerth et al., 2014) | N/A (Weckwerth et al., 2014) | | |
| 1 | *Providencia stuartii* | SCOM secretion (Weckwerth et al., 2009) | Not prevalent (Weckwerth et al., 2009) | N/A (Weckwerth et al., 2009) | | |
| 1 | *Selenomonas* spp. | Saliva (Zhang et al., 2022) | Not prevalent (Zhang et al., 2022) | N/A (Zhang et al., 2022) | | |
| 1 | *Shuttleworthia* spp. | Saliva (Zhang et al., 2022) | Not prevalent (Zhang et al., 2022) | N/A (Zhang et al., 2022) | | |
| 1 | *Solobacterium* spp. | Saliva (Zhang et al., 2022) | Not prevalent (Zhang et al., 2022) | N/A (Zhang et al., 2022) | | |
| 1 | *Tatumella ptyseos* | Blood (Adeyemo et al., 2013) | Not prevalent (Adeyemo et al., 2013) | N/A (Adeyemo et al., 2013) | | |
| 1 | *Tannerella* spp. | Saliva (Liu et al., 2016) | Not prevalent (Liu et al., 2016) | N/A (Liu et al., 2016) | | |
| 1 | *Treponema* spp. | Saliva (Zhang et al., 2022) | Not prevalent (Zhang et al., 2022) | N/A (Zhang et al., 2022) | | |
| 1 | *Yersinia enterocolitica* | Blood (Adeyemo et al., 2013) | Not prevalent (Adeyemo et al., 2013) | N/A (Adeyemo et al., 2013) | | |

**Supplementary Table S7. Bacterial phyla found in the oral environment of CL/P and non-CL/P subjects.**

| **Phylum Frequency** | **Phylum Name** | **Location** | **Prevalence in CL/P** | **Significant Difference** | |
| --- | --- | --- | --- | --- | --- |
|  |  |  |  | **Prone to non-CL/P** | **Prone to CL/P** |
| 4 | *Actinobacteria* | Cleft site (Rodrigues et al., 2021); Alveolar bone mucosa (Zhang et al., 2022); Saliva (Liu et al., 2016; Zhang et al., 2022) | Not prevalent (Liu et al., 2016; Rodrigues et al., 2021; Zhang et al., 2022) | N/A (Liu et al., 2016; Rodrigues et al., 2021; Zhang et al., 2022) | |
| 4 | *Bacteroidetes* | Cleft site (Rodrigues et al., 2021); Alveolar bone mucosa (Zhang et al., 2022); Saliva (Liu et al., 2016; Zhang et al., 2022) | Not prevalent (Liu et al., 2016; Rodrigues et al., 2021; Zhang et al., 2022) | N/A (Liu et al., 2016; Rodrigues et al., 2021; Zhang et al., 2022) | |
| 4 | *Firmicutes* | Cleft site (Rodrigues et al., 2021); Alveolar bone mucosa (Zhang et al., 2022); Saliva (Liu et al., 2016; Zhang et al., 2022) | Moderately prevalent (Rodrigues et al., 2021; Zhang et al., 2022); Somewhat prevalent (Liu et al., 2016) | N/A (Liu et al., 2016; Rodrigues et al., 2021; Zhang et al., 2022) | |
| 4 | *Fusobacterium* | Cleft site (Rodrigues et al., 2021); Alveolar bone mucosa (Zhang et al., 2022); Saliva (Liu et al., 2016; Zhang et al., 2022) | Not prevalent (Liu et al., 2016; Rodrigues et al., 2021; Zhang et al., 2022) | N/A (Liu et al., 2016; Rodrigues et al., 2021; Zhang et al., 2022) | |
| 4 | *Proteobacteria* | Cleft site (Rodrigues et al., 2021); Alveolar bone mucosa (Zhang et al., 2022); Saliva (Liu et al., 2016; Zhang et al., 2022) | Somewhat prevalent (Liu et al., 2016; Zhang et al., 2022); Not prevalent (Rodrigues et al., 2021) | N/A (Liu et al., 2016; Rodrigues et al., 2021; Zhang et al., 2022) | |
| 1 | *Mycoplasmatota* | Saliva (Liu et al., 2016) | Not prevalent (Liu et al., 2016) | N/A (Liu et al., 2016) | |
| 1 | *Patescibacteria* | Cleft site (Rodrigues et al., 2021) | Not prevalent (Rodrigues et al., 2021) | N/A (Rodrigues et al., 2021) | |
| 1 | *Synergistota* | Saliva (Liu et al., 2016) | Not prevalent (Liu et al., 2016) | N/A (Liu et al., 2016) | |

**Supplementary Table S8. *Candida* (fungal) species in CL/P and non-CL/P patients.**

| **Species Frequency** | **Species Name** | **Location** | **Prevalence in CL/P** | **Significant Difference** | | |
| --- | --- | --- | --- | --- | --- | --- |
|  |  |  |  | **Prone to non-CL/P** | **Prone to CL/P** | **Not Prone to Either** |
| 11 | *Candida albicans* | Teeth adjacent to the cleft (Machorowska-Pieniążek et al., 2013); Nose and/or oropharynx (Thomas et al., 2012); Soft palate and nasopharynx (Roode et al., 2022); Palatal cleft and nasopharynx (Roode and Bütow, 2018); Soft palate and nasopharynx (Mÿburgh and Bütow, 2009); Oral and buccal mucosa (Mattos et al., 2009); Hard palate (Yilmaz et al., 2020); Sublingual region, tongue dorsum, palate, buccal mucosa, and gingival margin (de Souza et al., 2022); Tongue dorsum and buccal and palatal mucosae (Rawashdeh et al., 2011); Palatal cleft (Silva et al., 2018); Oral rinse (Boriollo et al., 2022) | Highly prevalent (Mattos et al., 2009); Moderately prevalent (Rawashdeh et al., 2011; Boriollo et al., 2022); Not prevalent (Mÿburgh and Bütow, 2009; Thomas et al., 2012; Machorowska-Pieniążek et al., 2013; Roode and Bütow, 2018; Silva et al., 2018; Yilmaz et al., 2020; Roode et al., 2022) | N/A (Mattos et al., 2009; Mÿburgh and Bütow, 2009; Thomas et al., 2012; Machorowska-Pieniążek et al., 2013; Roode and Bütow, 2018; Silva et al., 2018; Yilmaz et al., 2020; Boriollo et al., 2022; Roode et al., 2022) | | |
|  |  |  |  | See *Candida* spp. (Rawashdeh et al., 2011) | | |
|  |  |  | See *Candida* spp*.* (de Souza et al., 2022) | | | |
| 5 | *Candida tropicalis* | Soft palate and nasopharynx (Roode et al., 2022); Palatal cleft and nasopharynx (Roode and Bütow, 2018); Sublingual region, tongue dorsum, palate, buccal mucosa, and gingival margin (de Souza et al., 2022); Palatal cleft (Silva et al., 2018); Oral rinse (Boriollo et al., 2022) | Not prevalent (Roode and Bütow, 2018; Silva et al., 2018; Boriollo et al., 2022; Roode et al., 2022) | N/A (Roode and Bütow, 2018; Silva et al., 2018; Boriollo et al., 2022; Roode et al., 2022) | | |
|  |  |  | See *Candida* spp. (de Souza et al., 2022) | | | |
| 6 | *Candida* spp. | Saliva (Durhan et al., 2019); Hard palate (Yilmaz et al., 2020); Sublingual region, tongue dorsum, palate, buccal mucosa, and gingival margin (de Souza et al., 2022); Tongue dorsum and buccal and palatal mucosae (Rawashdeh et al., 2011); Palatal cleft (Silva et al., 2018); Oral rinse (Boriollo et al., 2022) | Highly prevalent (Rawashdeh et al., 2011; Boriollo et al., 2022; de Souza et al., 2022); Moderately prevalent (Durhan et al., 2019); Somewhat prevalent (Silva et al., 2018); Not prevalent (Yilmaz et al., 2020) |  | (Rawashdeh et al., 2011; Boriollo et al., 2022; de Souza et al., 2022) | (Durhan et al., 2019) |
|  |  |  |  | N/A (Silva et al., 2018; Yilmaz et al., 2020) | | |
| 5 | *Candida krusei* | Soft palate and nasopharynx (Roode et al., 2022); Palatal cleft and nasopharynx (Roode and Bütow, 2018); Sublingual region, tongue dorsum, palate, buccal mucosa, and gingival margin (de Souza et al., 2022); Palatal cleft (Silva et al., 2018); Oral rinse (Boriollo et al., 2022) | Somewhat prevalent (Boriollo et al., 2022); Not prevalent (Roode and Bütow, 2018; Silva et al., 2018; Roode et al., 2022) | N/A (Roode and Bütow, 2018; Silva et al., 2018; Boriollo et al., 2022; Roode et al., 2022) | | |
|  |  |  | See *Candida* spp. (de Souza et al., 2022) | | | |
| 3 | *Candida kefyr* | Soft palate and nasopharynx (Roode et al., 2022); Palatal cleft and nasopharynx (Roode and Bütow, 2018); Tongue dorsum and buccal and palatal mucosae (Rawashdeh et al., 2011) | Not prevalent (Rawashdeh et al., 2011; Roode and Bütow, 2018; Roode et al., 2022) | N/A (Roode and Bütow, 2018; Roode et al., 2022) | | |
|  |  |  |  | See *Candida* spp. (Rawashdeh et al., 2011) | | |
| 3 | *Candida parapsilosis* | Soft palate and nasopharynx (Roode et al., 2022); Palatal cleft and nasopharynx (Roode and Bütow, 2018); Sublingual region, tongue dorsum, palate, buccal mucosa, and gingival margin (de Souza et al., 2022) | Not prevalent (Roode and Bütow, 2018; Boriollo et al., 2022; Roode et al., 2022) | N/A (Roode and Bütow, 2018; Boriollo et al., 2022; Roode et al., 2022) | | |
|  |  |  | See *Candida* spp. (de Souza et al., 2022) | | | |
| 2 | *Candida famata* | Soft palate and nasopharynx (Roode et al., 2022); Palatal cleft and nasopharynx (Roode and Bütow, 2018) | Not prevalent (Roode and Bütow, 2018; Roode et al., 2022) | N/A (Roode and Bütow, 2018; Roode et al., 2022) | | |
| 2 | *Candida glabrata* | Soft palate and nasopharynx (Roode et al., 2022); Tongue dorsum and buccal and palatal mucosae (Rawashdeh et al., 2011) | Not prevalent (Rawashdeh et al., 2011; Roode et al., 2022) | N/A (Roode et al., 2022) | | |
|  |  |  |  | See *Candida* spp. (Rawashdeh et al., 2011) | | |
| 2 | *Candida lusitaniae* | Soft palate and nasopharynx (Roode et al., 2022); Palatal cleft and nasopharynx (Roode and Bütow, 2018) | Not prevalent (Roode and Bütow, 2018; Roode et al., 2022) | N/A (Roode and Bütow, 2018; Roode et al., 2022) | | |
| 2 | non-*C. albicans* *Candida* spp. | Hard palate (Yilmaz et al., 2020); Oral rinse (Boriollo et al., 2022) | Not prevalent (Yilmaz et al., 2020; Boriollo et al., 2022) | N/A (Yilmaz et al., 2020; Boriollo et al., 2022) | | |
| 1 | *Candida dublinensis* | Soft palate and nasopharynx (Roode et al., 2022) | Not prevalent (Roode et al., 2022) | N/A (Roode et al., 2022) | | |

**Supplementary Table S9. Association of viruses (including viral infection of the mother during gestation) and CL/P.**

| **Virus Frequency** | **Virus Name** | **Association with CL/P** |
| --- | --- | --- |
| 6 | *Influenza* | Positive association (Acs et al., 2005; Métneki et al., 2005; Ács et al., 2020); no association (Rintala et al., 1983; Moro et al., 2013; Choi et al., 2019) |
| 2 | *Human Cytomegalovirus* | No association (Cerný et al., 1991; Divya et al., 2017) |
| 2 | *Respiratory syncytical virus* | No association (Rintala et al., 1983; Zachariah et al., 2011) |
| 1 | *Epstein-Barr virus* | No association (Cerný et al., 1991) |
| 1 | *Herpes simplex virus (orofacial herpes)* | Positive association (Métneki et al., 2005) |
| 1 | *Adenoviruses* | No association (Rintala et al., 1983) |
| 1 | *Coxsackie virus* | No association (Molnarova et al., 2002) |
| 1 | *Human Immunodeficiency Virus* | No association (James et al., 2014) |
| 1 | *Measles* | No association (Rintala et al., 1983) |
| 1 | *Mumps* | No association (Rintala et al., 1983) |
| 1 | *Parainfluenza* | No association (Rintala et al., 1983) |
| 1 | *Rubeola* | No association (Rintala et al., 1983) |
| 1 | *West Nile Virus* | No association (O'Leary et al., 2006) |
| 1 | *Zika virus* | No association (Rodriguez-Morales et al., 2018) |

**References**

Ács, L., Bányai, D., Nemes, B., Nagy, K., Ács, N., Bánhidy, F., et al. (2020). Maternal-related factors in the origin of isolated cleft palate-A population-based case-control study. *Orthod Craniofac Res* 23(2)**,** 174-180. doi: 10.1111/ocr.12361.

Acs, N., Bánhidy, F., Puhó, E., and Czeizel, A.E. (2005). Maternal influenza during pregnancy and risk of congenital abnormalities in offspring. *Birth Defects Res A Clin Mol Teratol* 73(12)**,** 989-996. doi: 10.1002/bdra.20195.

Adeyemo, W.L., Adeyemi, M.O., Ogunsola, F.T., Ogunlewe, M.O., Ladeinde, A.L., Mofikoya, B.O., et al. (2013). Prevalence and bacteriology of bacteremia associated with cleft lip and palate surgery. *J Craniofac Surg* 24(4)**,** 1126-1131. doi: 10.1097/SCS.0b013e31828016e8.

Antoszewska, J., Kawala, B., and Minch, L. (2010). Selected aspects of the oral environment in cleft palate patients--a problem evidently beyond dentists' scope. *Postepy Hig Med Dosw (Online)* 64**,** 659-664.

Arief, E.M., Mohamed, Z., and Idris, F.M. (2005). Study of viridans streptococci and Staphylococcus species in cleft lip and palate patients before and after surgery. *Cleft Palate Craniofac J* 42(3)**,** 277-279. doi: 10.1597/04-083r.1.

Bokhout, B., van Loveren, C., Hofman, F.X., Buijs, J.F., van Limbeek, J., and Prahl-Andersen, B. (1996). Prevalence of Streptococcus mutans and lactobacilli in 18-month-old children with cleft lip and/or palate. *Cleft Palate Craniofac J* 33(5)**,** 424-428. doi: 10.1597/1545-1569_1996_033_0424_posmal_2.3.co_2.

Boriollo, M.F.G., Oliveira, M.C., Bassinello, V., Aníbal, P.C., da Silva, T.A., da Silva, J.J., et al. (2022). Candida species biotypes and polyclonality of potentially virulent Candida albicans isolated from oral cavity of patients with orofacial clefts. *Clin Oral Investig* 26(3)**,** 3061-3084. doi: 10.1007/s00784-021-04290-z.

Bos, M., Hopman, J., Stuiver, M.M., and Voss, A. (2016). Decolonisation of meticillin-resistant Staphylococcus aureus (MRSA) carriage in adopted children with cleft lip and palate. *J Glob Antimicrob Resist* 7**,** 28-33. doi: 10.1016/j.jgar.2016.07.001.

Cerný, M., Fára, M., and Hrivnáková, J. (1991). Aetiological, modifying and lethal factors in cleft lip and palate. *Acta Chir Plast* 33(2)**,** 72-86.

Chaudhari, P.K., Kharbanda, O.P., Chaudhry, R., Pandey, R.M., Chauhan, S., Bansal, K., et al. (2021). Factors Affecting High Caries Risk in Children With and Without Cleft Lip and/or Palate: A Cross-Sectional Study. *Cleft Palate Craniofac J* 58(9)**,** 1150-1159. doi: 10.1177/1055665620980206.

Cheng, L.L., Moor, S.L., Kravchuk, O., Meyers, I.A., and Ho, C.T. (2007). Bacteria and salivary profile of adolescents with and without cleft lip and/or palate undergoing orthodontic treatment. *Aust Dent J* 52(4)**,** 315-321. doi: 10.1111/j.1834-7819.2007.tb00508.x.

Choi, W.S., Choi, M.J., Noh, J.Y., Song, J.Y., Kim, W.J., Park, D.W., et al. (2019). Clinical and economic analysis of the 2009 H1N1 influenza pandemic among pregnant Korean women. *Korean J Intern Med* 34(5)**,** 1136-1144. doi: 10.3904/kjim.2017.107.

Chuo, C.B., and Timmons, M.J. (2005). The bacteriology of children before primary cleft lip and palate surgery. *Cleft Palate Craniofac J* 42(3)**,** 272-276. doi: 10.1597/03-108.1.

Cildir, S.K., Sandalli, N., Nazli, S., Alp, F., and Caglar, E. (2012). A novel delivery system of probiotic drop and its effect on dental caries risk factors in cleft lip/palate children. *Cleft Palate Craniofac J* 49(3)**,** 369-372. doi: 10.1597/10-035.

Cocco, J.F., Antonetti, J.W., Burns, J.L., Heggers, J.P., and Blackwell, S.J. (2010). Characterization of the nasal, sublingual, and oropharyngeal mucosa microbiota in cleft lip and palate individuals before and after surgical repair. *Cleft Palate Craniofac J* 47(2)**,** 151-155. doi: 10.1597/08-187_1.

Costa, B., Lima, J.E., Gomide, M.R., and Rosa, O.P. (2003). Clinical and microbiological evaluation of the periodontal status of children with unilateral complete cleft lip and palate. *Cleft Palate Craniofac J* 40(6)**,** 585-589. doi: 10.1597/01-083.

de Soet, J.J., Bokhout, B., Buijs, J.F., van Loveren, C., de Graaff, J., and Prahl-Andersen, B. (1998). Transmission of mutans streptococci between mothers and children with cleft lip and/or palate. *Cleft Palate Craniofac J* 35(5)**,** 460-464. doi: 10.1597/1545-1569_1998_035_0460_tomsbm_2.3.co_2.

de Souza, P.T.D.R., Gonçalves-Wilhelmsen, N.C.V., Rosa, R.T., Correia, C.F.K.N., Pereira, T.M., Kitahara, A.B.P., et al. (2022). Oral Colonization and Virulence Factors of *Candida* spp. in Babies With Cleft Palate. *Cleft Palate Craniofac J* 59(8)**,** 1056-1063. doi: 10.1177/10556656211030437.

Divya, D.V., Prasad, M.G.S., Radhakrishna, A.N., Reddy, S.P., Pratyusha, K., Kumar, K.V.K.S., et al. (2017). The Serological Evidence of Cytomegalovirus Infection as a Potent Aetiological Factor for Cleft Lip/Palate, Mental Retardation and Deafness. *J Clin Diagn Res* 11(6)**,** ZC51-ZC54. doi: 10.7860/JCDR/2017/25118.10067.

Durhan, M.A., Topcuoglu, N., Kulekci, G., Ozgentas, E., and Tanboga, I. (2019). Microbial Profile and Dental Caries in Cleft Lip and Palate Babies Between 0 and 3 Years Old. *Cleft Palate Craniofac J* 56(3)**,** 349-356. doi: 10.1177/1055665618776428.

Funahashi, K., Shiba, T., Watanabe, T., Muramoto, K., Takeuchi, Y., Ogawa, T., et al. (2019). Functional dysbiosis within dental plaque microbiota in cleft lip and palate patients. *Prog Orthod* 20(1)**,** 11. doi: 10.1186/s40510-019-0265-1.

Hassani, H., Chen, J.W., Zhang, W., and Hamra, W. (2020). Comparison of Microbial Activity Among Infants With or Without Using Presurgical Nasoalveolar Molding Appliance. *Cleft Palate Craniofac J* 57(6)**,** 762-769. doi: 10.1177/1055665620908150.

Iurovschi, R., Joaquim, C.R., de Faveri, M., de Miranda, T.S., Feres, M., and de Figueiredo, L.C. (2020). Evaluation of the Microbiological Profile of Alveolar Residual Screws and Cleft-Adjacent Teeth in Individuals With Complete Unilateral Fissures. *Cleft Palate Craniofac J* 57(10)**,** 1182-1189. doi: 10.1177/1055665620945568.

James, A., Oluwatosin, B., Njideka, G., Babafemi, Benjamin, O.G., Olufemi, D., et al. (2014). Cleft Palate In HIV-Exposed Newborns Of Mothers On Highly Active Antiretroviral Therapy. *Oral Surg* 7(Suppl 1)**,** 102-106. doi: 10.1111/ors.12117.

Jousimies-Somer, H., Grénman, R., and Rintala, A. (1986). Bacteriological investigation of secretory otitis media in children with cleft palate. *Scand J Plast Reconstr Surg* 20(3)**,** 297-302. doi: 10.3109/02844318609004490.

Liu, L., Zhang, Q., Lin, J., Ma, L., Zhou, Z., He, X., et al. (2016). Investigating Oral Microbiome Profiles in Children with Cleft Lip and Palate for Prognosis of Alveolar Bone Grafting. *PLoS One* 11(5)**,** e0155683. doi: 10.1371/journal.pone.0155683.

Lucas, V.S., Gupta, R., Ololade, O., Gelbier, M., and Roberts, G.J. (2000). Dental health indices and caries associated microflora in children with unilateral cleft lip and palate. *Cleft Palate Craniofac J* 37(5)**,** 447-452. doi: 10.1597/1545-1569_2000_037_0447_dhiaca_2.0.co_2.

Machorowska-Pieniążek, A., Mertas, A., Skucha-Nowak, M., Tanasiewicz, M., and Morawiec, T. (2017). A Comparative Study of Oral Microbiota in Infants with Complete Cleft Lip and Palate or Cleft Soft Palate. *Biomed Res Int* 2017**,** 1460243. doi: 10.1155/2017/1460243.

Machorowska-Pieniążek, A., Morawiec, T., Mertas, A., Tanasiewicz, M., Dziedzic, A., and Król, W. (2013). Influence of propolis on hygiene, gingival condition, and oral microflora in patients with cleft lip and palate treated with fixed orthodontic appliances. *Evid Based Complement Alternat Med* 2013**,** 183915. doi: 10.1155/2013/183915.

Mattos, B.S., Sousa, A.A., Magalhães, M.H., André, M., and Brito E Dias, R. (2009). Candida albicans in patients with oronasal communication and obturator prostheses. *Braz Dent J* 20(4)**,** 336-340. doi: 10.1590/s0103-64402009000400013.

Métneki, J., Puhó, E., and Czeizel, A.E. (2005). Maternal diseases and isolated orofacial clefts in Hungary. *Birth Defects Res A Clin Mol Teratol* 73(9)**,** 617-623. doi: 10.1002/bdra.20177.

Molnarova, A., Petrovicova, A., Fedeles, J., Bopegamage, S., and Horakova, E. (2002). Coxsackie viral infection and orofacial cleft. *Bratisl Lek Listy* 103(10)**,** 365-367.

Mombelli, A., Brägger, U., and Lang, N.P. (1992). Microbiota associated with residual clefts and neighboring teeth in patients with cleft lip, alveolus, and palate. *Cleft Palate Craniofac J* 29(5)**,** 463-469. doi: 10.1597/1545-1569_1992_029_0463_mawrca_2.3.co_2.

Moro, P.L., Museru, O.I., Broder, K., Cragan, J., Zheteyeva, Y., Tepper, N., et al. (2013). Safety of influenza A (H1N1) 2009 live attenuated monovalent vaccine in pregnant women. *Obstet Gynecol* 122(6)**,** 1271-1278. doi: 10.1097/AOG.0000000000000010.

Mÿburgh, H.P., and Bütow, K.W. (2009). Cleft soft palate reconstruction: prospective study on infection and antibiotics. *Int J Oral Maxillofac Surg* 38(9)**,** 928-932. doi: 10.1016/j.ijom.2009.04.022.

Narinesingh, S.P., Whitby, D.J., and Davenport, P.J. (2011). Moraxella catarrhalis: an unrecognized pathogen of the oral cavity? *Cleft Palate Craniofac J* 48(4)**,** 462-464. doi: 10.1597/09-054.

O'Leary, D.R., Kuhn, S., Kniss, K.L., Hinckley, A.F., Rasmussen, S.A., Pape, W.J., et al. (2006). Birth outcomes following West Nile Virus infection of pregnant women in the United States: 2003-2004. *Pediatrics* 117(3)**,** e537-545. doi: 10.1542/peds.2005-2024.

Parapanisiou, V., Gizani, S., Makou, M., and Papagiannoulis, L. (2009). Oral health status and behaviour of Greek patients with cleft lip and palate. *Eur Arch Paediatr Dent* 10(2)**,** 85-89. doi: 10.1007/BF03321606.

Passinato Gheller, S.A., Porto, A.N., Borba, A.M., Veiga, K.A., and Aranha, A.M.F. (2021). Periodontal Findings in Children and Adolescents with Cleft Lip and/or Palate: A Case-Control Study. *Pediatr Dent* 43(2)**,** 133-139.

Perdikogianni, H., Papaioannou, W., Nakou, M., Oulis, C., and Papagiannoulis, L. (2009). Periodontal and microbiological parameters in children and adolescents with cleft lip and /or palate. *Int J Paediatr Dent* 19(6)**,** 455-467. doi: 10.1111/j.1365-263X.2009.01020.x.

Quirynen, M., Dewinter, G., Avontroodt, P., Heidbüchel, K., Verdonck, A., and Carels, C. (2003). A split-mouth study on periodontal and microbial parameters in children with complete unilateral cleft lip and palate. *J Clin Periodontol* 30(1)**,** 49-56. doi: 10.1034/j.1600-051x.2003.300108.x.

Ramdial, S., and Madaree, A. (2019). The spectrum of intraoral bacteria seen in patients with cleft palates in an African setting. *Microbiologyopen* 8(4)**,** e00679. doi: 10.1002/mbo3.679.

Rawashdeh, M.A., Ayesh, J.A., and Darwazeh, A.M. (2011). Oral candidal colonization in cleft patients as a function of age, gender, surgery, type of cleft, and oral health. *J Oral Maxillofac Surg* 69(4)**,** 1207-1213. doi: 10.1016/j.joms.2010.02.044.

Rennie, A., Treharne, L.J., and Richard, B. (2009). Throat swabs taken on the operating table prior to cleft palate repair and their relevance to outcome: a prospective study. *Cleft Palate Craniofac J* 46(3)**,** 275-279. doi: 10.1597/08-082.1.

Rintala, A., Pönkä, A., Sarna, S., and Stegars, T. (1983). Cleft lip and palate in Finland in 1948-75: correlations to infections, seasonal and yearly variations. *Scand J Plast Reconstr Surg* 17(3)**,** 197-201. doi: 10.3109/02844318309013119.

Ritthagol, W., Saetang, C., and Teanpaisan, R. (2014). Effect of Probiotics Containing Lactobacillus paracasei SD1 on Salivary Mutans Streptococci and Lactobacilli in Orthodontic Cleft Patients: A Double-Blinded, Randomized, Placebo-Controlled Study. *Cleft Palate Craniofac J* 51(3)**,** 257-263. doi: 10.1597/12-243.

Rodrigues, R., Chung, A.P., Mortensen, M.S., Fernandes, M.H., Monteiro, A.B., Furfuro, R., et al. (2021). Temporal oral microbiome changes with brushing in children with cleft lip and palate. *Heliyon* 7(3)**,** e06513. doi: 10.1016/j.heliyon.2021.e06513.

Rodriguez-Morales, A.J., Cardona-Ospina, J.A., Ramirez-Jaramillo, V., Gaviria, J.A., González-Moreno, G.M., Castrillón-Spitia, J.D., et al. (2018). Diagnosis and outcomes of pregnant women with Zika virus infection in two municipalities of Risaralda, Colombia: Second report of the ZIKERNCOL study. *Travel Med Infect Dis* 25**,** 20-25. doi: 10.1016/j.tmaid.2018.06.006.

Roode, G.J., and Bütow, K.W. (2018). A Descriptive Study of Chlorhexidine as a Disinfectant in Cleft Palate Surgery. *Clin Med Res* 16(1-2)**,** 9-15. doi: 10.3121/cmr.2018.1385.

Roode, G.J., Bütow, K.W., and Naidoo, S. (2017). Preoperative evaluation of micro-organisms in non-operated cleft in soft palate: impact on use of antibiotics. *Br J Oral Maxillofac Surg* 55(2)**,** 127-131. doi: 10.1016/j.bjoms.2016.09.018.

Roode, G.J., Bütow, K.W., and Naidoo, S. (2022). Microbial contamination profile change over a 4-year period in nonoperated cleft soft palate. *J Appl Microbiol* 132(1)**,** 665-674. doi: 10.1111/jam.15193.

Shashni, R., Goyal, A., Gauba, K., Utreja, A.K., Ray, P., and Jena, A.K. (2015). Comparison of risk indicators of dental caries in children with and without cleft lip and palate deformities. *Contemp Clin Dent* 6(1)**,** 58-62. doi: 10.4103/0976-237X.149293.

Silva, J.J.D., Silva, T.A.D., Almeida, H., Rodrigues Netto, M.F., Cerdeira, C.D., Höfling, J.F., et al. (2018). Candida species biotypes in the oral cavity of infants and children with orofacial clefts under surgical rehabilitation. *Microb Pathog* 124**,** 203-215. doi: 10.1016/j.micpath.2018.08.042.

Sundell, A.L., Ullbro, C., Dahlén, G., Marcusson, A., and Twetman, S. (2018). Salivary microbial profiles in 5-year old children with oral clefts: a comparative study. *Eur Arch Paediatr Dent* 19(1)**,** 57-60. doi: 10.1007/s40368-018-0326-z.

Sundell, A.L., Ullbro, C., Marcusson, A., and Twetman, S. (2015). Comparing caries risk profiles between 5- and 10- year-old children with cleft lip and/or palate and non-cleft controls. *BMC Oral Health* 15**,** 85. doi: 10.1186/s12903-015-0067-x.

Thomas, G.P., Sibley, J., Goodacre, T.E., and Cadier, M.M. (2012). The value of microbiological screening in cleft lip and palate surgery. *Cleft Palate Craniofac J* 49(6)**,** 708-713. doi: 10.1597/11-063.

Tuna, E.B., Topçuoglu, N., Ilhan, B., Gençay, K., and Kulekçi, G. (2008). Staphylococcus aureus transmission through oronasal fistula in children with cleft lip and palate. *Cleft Palate Craniofac J* 45(5)**,** 477-480. doi: 10.1597/06-247.1.

van Loveren, C., Buijs, J.F., Bokhout, B., Prahl-Andersen, B., and Ten Cate, J.M. (1998). Incidence of mutans streptococci and lactobacilli in oral cleft children wearing acrylic plates from shortly after birth. *Oral Microbiol Immunol* 13(5)**,** 286-291. doi: 10.1111/j.1399-302x.1998.tb00709.x.

Vieira, N.A., Borgo, H.C., da Silva Dalben, G., Bachega, M.I., and Pereira, P.C. (2013). Evaluation of fecal microorganisms of children with cleft palate before and after palatoplasty. *Braz J Microbiol* 44(3)**,** 835-838. doi: 10.1590/s1517-83822013000300026.

Weckwerth, P.H., de Magalhães Lopes, C.A., Duarte, M.A., Weckwerth, A.C., Martins, C.H., Neto, D.L., et al. (2009). Chronic suppurative otitis media in cleft palate: microorganism etiology and susceptibilities. *Cleft Palate Craniofac J* 46(5)**,** 461-467. doi: 10.1597/08-144.1.

Weckwerth, P.H., de Mattias Franco, A.T., de Magalhães Lopes, C.A., Santos, F.D., Weckwerth, A.C., Vivan, R.R., et al. (2014). Bacterial pathogens related to chronic suppurative otitis media in individuals with cleft palate: bacteriological culture and polymerase chain reaction. *Cleft Palate Craniofac J* 51(2)**,** 145-153. doi: 10.1597/11-325.

Yilmaz, H.N., Hatipoglu, S., Erdem, B., Can, B., and Kadir, T. (2020). Adherence frequency of CANDIDA ALBICANS on nasoalveolar molding (NAM) appliances. *J Stomatol Oral Maxillofac Surg* 121(5)**,** 473-477. doi: 10.1016/j.jormas.2020.08.005.

Zachariah, P., Ruttenber, M., and Simões, E.A. (2011). Hospitalizations due to respiratory syncytial virus in children with congenital malformations. *Pediatr Infect Dis J* 30(5)**,** 442-445. doi: 10.1097/INF.0b013e318201813b.

Zhang, K., Zhou, X., Qin, J., Zhang, W., Pan, Y., Wang, H., et al. (2022). Dynamic Change in Oral Microbiota of Children With Cleft Lip and Palate After Alveolar Bone Grafting. *Cleft Palate Craniofac J* 59(11)**,** 1352-1360. doi: 10.1177/10556656211044396.

Zhang, M., Wang, R., Liao, Y., Buijs, M.J., and Li, J. (2016). Profiling of Oral and Nasal Microbiome in Children With Cleft Palate. *Cleft Palate Craniofac J* 53(3)**,** 332-338. doi: 10.1597/14-162.
